# Phylogeography of the veined squid, *Loligo forbesii,* in European waters

**DOI:** 10.1038/s41598-022-11530-z

**Published:** 2022-05-12

**Authors:** Anika Göpel, Daniel Oesterwind, Christopher Barrett, Rita Cannas, Luis Silva Caparro, Pierluigi Carbonara, Marilena Donnaloia, Maria Cristina Follesa, Angela Larivain, Vladimir Laptikhovsky, Evgenia Lefkaditou, Jean-Paul Robin, Maria Begoña Santos, Ignacio Sobrino, Julio Valeiras, Maria Valls, Hugo C. Vieira, Kai Wieland, Ralf Bastrop

**Affiliations:** 1 Thünen Institute of Baltic Sea Fisheries, Alter Hafen Süd 2, 18069 Rostock, Germany; 2 Institute of Biological Sciences, University of Rostock, Albert-Einstein-Str. 3, 18059 Rostock, Germany; 3 Cefas Laboratory, Pakefield Rd, Lowestoft, NR33 0HT UK; 4 Hellenic Centre for Marine Research, Institute of Marine Biological Resources and Inland Waters, 576 SideRD Vouliagmenis Ave, 16452 Athens, Greece; 5 Technical University of Denmark, National Institute of Aquatic Resources, Nordsøen Forskerpark, Willemoesvej 2, 9850 Hirtshals, Denmark; 6 University of Caen Normandy, CS 14032, 14032 Caen Cedex 05, France; 7 Centre Oceanográfic de les Balears s/n, Instituto Español de Oceanografía (IEO), 07015 Palma, Spain; 8 Department of Life and Environmental Sciences, University of Cagliari, Cagliari, Italy; 9 COISPA Tecnologia & Ricerca, Via dei Trulli, 18-20, Bari, Italy; 10 Centro Oceanográfico de Cádiz, Instituto Español de Oceanografía, Puerto Pesquero, Muelle de Levante S/N, 11006 Cádiz, Spain; 11 Centro Oceanográfico de Vigo, Instituto Español de Oceanografía (IEO), Subida a Radio Faro, 50, 36390 Vigo, Spain; 12 CESAM - Centre for Environmental and Marine Studies, Department of Biology, University of Aveiro, Campus de Santiago, 3810-193 Aveiro, Portugal

**Keywords:** Ecology, Evolution, Genetics, Zoology, Ecology

## Abstract

The veined squid, *Loligo forbesii* Steenstrup, 1856, occurs at the European Shelf areas including the Azores and represents a valuable resource for the European commercial fishery in the North East Atlantic. However, very little is known about its population structure and phylogeography. This lack of knowledge also impedes the development of sustainable fishery management for this species. The present study combined the use of two types of markers that retrieve patterns of gene flow in different time spans; the analysis of 16 nuclear microsatellites and sequencing of the mitochondrial cytochrome oxidase subunit I (COI). Whereas the high mutation rate of microsatellites allows the description of recent patterns of connectivity in species, the lower mutation rate of COI provides phylogeographic patterns on a longer timescale. A total of 347 individuals of *L. forbesii* were investigated from nearly the entire distribution range of the species, including the North East Atlantic Shelf, the Azores and the Mediterranean. Individuals from the Western and Eastern Mediterranean Sea have never been included in a genetic study before. We were able to analyse COI sequences from all 12 sampling areas and define three clades of *L. forbesii.* Due to our large sampling area, we are presenting 13 COI-haplotypes that were previously unknown. The microsatellite analysis does not include the Azores but three main clades could be identified at the remaining 11 sampling sites. Low F_ST_ values indicate gene flow over large geographical distances. However, the genetically significant differences and an additional slight grouping in the microsatellite structure reveal that geographical barriers seem to influence the population structure and reduce gene flow. Furthermore, both markers provide strong evidence that the observed phylogeographic pattern reflects the geographical history of the Azores and the Mediterranean Sea.

## Introduction

Within the last decades biomass of various cephalopod populations and commercial catches increased world-wide^1^. Especially in the Atlantic Ocean, the commercial importance of the loliginids has grown^2,3^. Loliginids landings reached about 12,000 t^3^ in 2017 showing a fivefold increase between 2000 and 2017 in the entire Northeast Atlantic, which illustrates the growing socio-economic importance. Among the loliginids, the veined squid *Loligo forbesii* Steenstrup, 1856 is one of the most important species for the European fishery^3^. This species is neritic, associated to the shelf and equally distributed on the lower shelf (80–200 m) and the upper slope (200–500 m) in the northern Mediterranean^4^. In the North East Atlantic it occurs from the North Sea and UK waters to the Canary Islands and Azores^2^. This opportunistic predator is characterized by high growth rates. During its 12–16 months of life span, it can reach a dorsal mantle length of up to 900 mm^5,6^. As a semelparous species *L. forbesii* spawns only once in its lifetime^2^.

In most regions *L. forbesii* is unregulated fished as bycatch, but European target fisheries exist in the English Channel, Scottish and Portuguese waters^3^ and recreational fishery develops in UK and Norway. First prediction models for *L. forbesii* have been developed^7^, but up to now it has been difficult to develop a sustainable fishery management for this valuable resource due to knowledge gaps regarding population structure^8^, life cycle and spawning areas^9^. Here, we will focus on the first aspect and will shed new light on the genetic population structure of *L. forbesii*.

The mitochondrial cytochrome-c-oxidase I (COI) gene is one of the most commonly used markers for molecular systematics and is often used to infer phylogeographic patterns^10^. However, to elucidate the complex population structure of *L. forbesii*, allozymes and mitochondrial DNA sequences were found apparently unsuitable because of their extremely low levels of genetic variability^11,12^. In such cases, a more appropriate approach is the analysis of microsatellites. Microsatellites are short repetitive sequences in the nuclear genome showing high mutation rates and multiple alleles^13^, allowing a powerful analysis of recent patterns of population connectivity. Shaw et al.^12^ found a higher genetic variability by using microsatellites for *L. forbesii* compared with previous studies using allozyme and mitochondrial DNA (mtDNA) markers, and were able to identify subtle genetic differentiation between species occurring at the European shelf seas (Scotland to Northern Spain) and offshore population including the Azores, Rockall and Faroes. They suggest that water depth and isolating current regimes are responsible for the genetic differences between offshore and onshore areas.

To shed new light on the genetic structure and phylogeography of *L. forbesii* we performed a mitochondrial COI and nuclear microsatellite analysis using the same dataset. In comparison to Shaw et al.^12^ we were able to geographically expand the study area to almost the entire distribution range of *L. forbesii* including the North-East Atlantic (from the North Sea to the Gulf of Cadiz, including the Azores) and the Mediterranean Sea (from the Balearic to the Aegean Sea). The new findings in population structure, gene flow and habitat connectivity of *L. forbesii* might have a substantial influence on the management of this species’ fisheries, as they provide new information regarding the identification of different appropriate management units, so called “stocks”^14^.

## Material and methods

### Sampling

**Figure 1:**
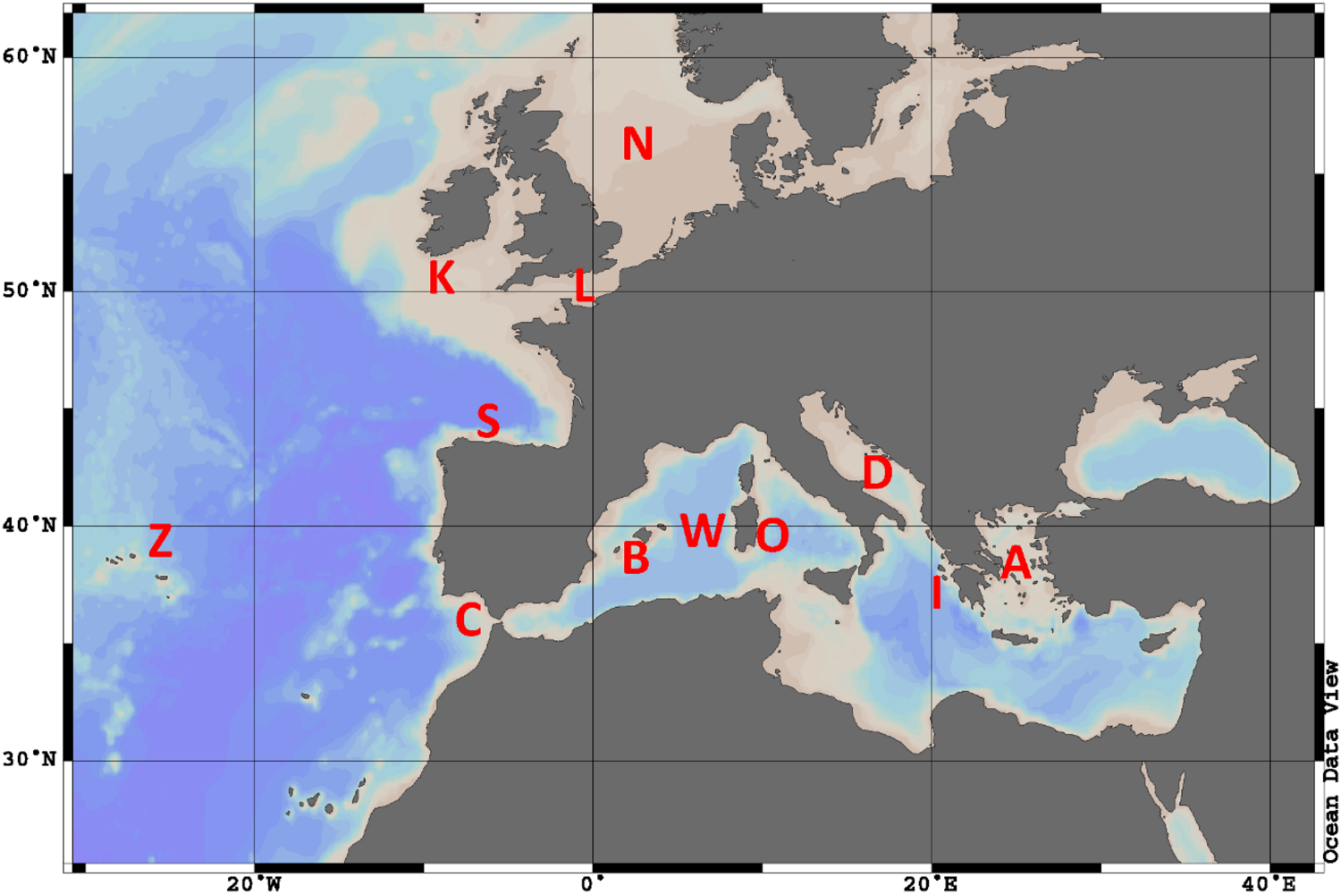
Sampled areas: A = Aegean Sea (19 individuals), B = Balearic Sea (28), C = Gulf of Cadiz (20), D = South Adriatic Sea (15), I = East Ionian Sea (30), K = Celtic Sea (19), L = English Channel (30), N = North Sea (64), O = east coast of Sardinia (30), S = Bay of Biscay (24), W = west coast of Sardinia (21), Z = Azores (47) (Ocean Data View version 4.6.2).

*Loligo forbesii* was sampled at 12 European areas, including shelf and upper slope areas and the Azores in 2019 (Fig. 1), resulting in a total of 347 analysed individuals (see Supplementary Table S1 online). Squids were trawled during different research cruises and frozen on board for processing on land. Additional individuals were collected at commercial markets.

From each individual, a pea-sized piece of muscle tissue was cut off the mantle and transferred into 96% undenatured ethanol. Ethanol was changed after 2 and 24 h if possible, to extract all water from the tissue and preserve the DNA. Samples, excluding North Sea samples which were prepared at the Thünen Institute of Baltic Sea Fisheries (TI-OF), were sent to the TI-OF, alcohol was changed once more and stored until the beginning of the genetic analysis at University of Rostock, where DNA was extracted using “innuPrepMini Kit” (Analytik Jena, Germany) following the manufacturer’s standard protocol.

### Ethical statement

*Loligo forbesii* is not protected under any legislation and not considered threatened or endangered. Atlantic samples were collected as bycatch during ICES coordinated international fishing trawl surveys (International Bottom Trawl Survey: North Sea, Bay of Biscay; Spanish Gulf of Cadiz Bottom Trawl Survey: Cadiz) and CEFAS Otter Trawl Survey (Celtic Sea) or at the fish market (English Channel). *L. forbesii* samples from the Mediterranean were bycatch from the International bottom trawl survey in the Mediterranean (MEDITS) except samples from the Balearic Sea which were collected from a commercial vessel. Azorean samples have been collected by the artisanal jigging fishery. All individuals were already dead when they were handed over to collect the tissue samples for our study and therefore handled in accordance with relevant guidelines and regulations. Fishing licenses for all surveys and trips were available.

### Population genetics via microsatellites

Population genetics of 300 individuals from eleven sampling areas were performed via microsatellite analysis at the University of Rostock using 16 specific primers (Lfor1–Lfor16) for *L. forbesii*^15,16^. Specimens from the Azores were not included in this analysis, because (1) we received the samples only 1 year after completion of the microsatellite analysis and (2) Shaw et al.^12^ already showed the uniqueness of *L. forbesii* in this area based on microsatellites. All primers were marked with a fluorescent dye. The master mix for the PCR included 0.5 µL of both primers (10 µM), 1.0 µL dNTPs (10 mM, 2.5 mM each), 1.0 µL 10 × Buffer, 0.085 µL Taq-Polymerase (5 units/µL) and between 0.8 and 1.2 µL MgCL_2_ (25 mM), referring to the primer. This was filled up to a volume of 9 µL with water. For the PCR reaction 1 µL DNA isolate was added. The reaction conditions followed the description of Shaw et al.^12^. Next, the concentration of DNA was estimated using calibrated gel electrophoresis and diluted accordingly. The diluted PCR product was added to 9.8 µL HiDi™ formamide and 0.02 µL size standard GeneScanTM LIZ™ (Applied Biosystems, Foster City, California, USA). The samples were denatured at 96 °C for 4 min and fragments were size-separated in a capillary sequencer (Hitachi 3130xl Genetic Analyzer, Applied Biosystems, Foster City, California, USA). If the signal in the electropherogram was too weak, the whole procedure was repeated up to three times.

The recorded electropherograms were evaluated using “Geneious Prime” (version 2019.04, Biomatters, New Zealand). The evaluation followed the guidelines of Butler^17^. Thereafter, the software performed the allele binning and the length of the different alleles was determined.

For the statistical evaluation several programs were applied. The “Microsatellite Toolkit” by Park^18^ calculated the number of alleles per locus as well as the expected heterozygosity^19^ and the observed heterozygosity^20^. Furthermore, statistics on linkage disequilibrium and Hardy–Weinberg Principle^21^ were performed with “GenePop”^22,23^. “HP RARE”^24^ was used to determine allelic richness and private allelic richness, based on 15 individuals. The calculation and the pairwise comparison of the F_ST_ values were performed in “Arlequin Ver 3.5”^25^. Cluster analyses based on the microsatellite data were conducted with “STRUCTURE” version 2.3.44^26^. All settings were selected according to the standard settings for microsatellite data as recommended by Gilbert et al.^27^ and Porras-Hurtado et al.^28^. For a K-value from 1 to 10 a total of 25 iterations were calculated. Next, the best K-value was calculated with the ΔK method^29^, using the online software “Clumpak” (http://clumpak.tau.ac.il/)^30^.

### Phylogeography via COI-sequencing

To describe the phylogeography of *L. forbesii*, 218 individuals from all 12 sampling areas were analysed. The primers LCO1490 und HCO2198^31^ were used to amplify a 648 bp long part of the mitochondrial cytochrome oxidase I (COI) gene. For samples with poor DNA quality, internal primers (see Supplementary Table S2 online) were designed to bisect the sequence into two shorter parts. These two internal sequences were then aligned to generate the full sequence. The PCR-Mix contained: 3 µL DNA-isolate, 3 µL dNTPs (10 mM, 2.5 mM each), 3 µL of each primer (10 µM), 3 µL 10 × Buffer, 4.8 µL MgCl_2_ (25 mM), 0.21 µL Taq-Polymerase (5 units/µL) and 9.99 µL water. The PCR was conducted in 38 cycles: 30 s at 94 °C, 30 s at 55 °C, 60 s at 72 °C and finally 300 s at 72 °C.

For the sequencing, the “BigDye Terminator v1.1 Kit” (Applied Biosystems, Foster City, California, USA) was used. Cycle sequencing products were analysed by using capillary separation on an ABI Genetic Analyzer 3130 xl (Applied Biosystems/Hitachi). All products were sequenced in both directions. The sequences obtained were analysed with the software “CEQ8000 Genetic Analysis System” (Version 9.0.25, Beckman Coulter GmbH, Germany) and an alignment of 249 sequences was created (“BioEdit” version 7.2.5.^32^) of which 28 were retrieved from GenBank^32–40^. The phylogenetic analyses (Maximum Likelihood, ML) was performed using “MEGA” version 6^41^ with 1000 bootstraps. We determined the best fitted model of the ML method with “MEGA 6” on the basis of the Akaike Information Criterion (AIC), TN93 + G + I (Fig. 5).

“MrBayes 3.2.7”^42^ was used for the Bayesian Inference (BI) method. Two independent runs with four chains were performed for 2 million generations using the Markov Chain Monte Carlo (MCMC) method. Calculations of the consensus tree, including clade posterior probability (PP), were conducted based on the trees sampled after the chains converged using “Tracer 1.7”^43^. The first 25% were discarded as burn-in. We determined the best fitted model of the BI method with “Modeltest”, implemented in “MEGA 6”, on the basis of the Bayesian information criterion (BIC), T92 + G.

Finally, a haplotype network with 249 individuals was calculated (Median-Joining Method^44^) using “Network 5.0.1.0” and its standard settings (Fluxus Technology, Suffolk, UK). Additionally, a second haplotype network was calculated containing 313 individuals with shorter sequences (432 bp), because some individuals yielded incomplete or shorter sequences (see Supplementary Fig. S1 online). Gene flow (Nm) and F_ST_ values for the COI-gene were calculated using “DnaSP v5”^45^ (see Supplementary Table S3 online).

All maps were created with “Ocean Data View” version 4.6.2^46^.

## Results

### Microsatellite analysis

In total, 16 microsatellite loci were tested for 300 individuals of *L. forbesii*. The locus Lfor7 showed up to eight peaks per individual. The loci Lfor14 and Lfor15 showed additional polymorphic amplification products outside the expected size range. Consequently, all three loci were excluded from statistical analyses. In addition, 4.92% of the samples could not be analysed.

The remaining loci showed multiple alleles, except Lfor9 which was monomorphic. The number of alleles per locus ranged from 11 (Lfor2) to 49 (Lfor12). The test of linkage disequilibrium showed that all loci are inherited independently. Some deviations of expected and observed heterozygosity were found to be significant. On average across all loci for each sampled area, only non-significant deviations were determined, meaning the Hardy–Weinberg equilibrium could not be rejected.

The average private allelic richness (Table 1) across all loci ranged from 0.06 (West-Sardinia) to 1.15 (Celtic Sea) and revealed high gene flow (for more details see Appendix S4).

**Table 1:** Overview of the average values of the microsatellite analysis per respective sampling area; allelic richness and private allelic richness (pAR) based on 15 individuals; Ho = observed heterozygosity; He = expected Heterozygosity; information for separate loci in Supplementary Table S4 online.

	North Sea	English Channel	Celtic Sea	Bay of Biscay	Gulf of Cadiz	Balearic Sea	West-Sardinia	East-Sardinia	Adriatic Sea	Ionian Sea	Aegean Sea
Sample size	64	30	19	24	20	28	21	30	15	30	19
Mean number of alleles	16.62	13.69	13.17	12.36	10.85	12.58	9.92	11.67	9.36	10.85	10.38
Allelic richness	11.460	11.480	12.670	13.600	10.570	10.170	9.080	9.830	12.580	9.470	10.430
pAR	0.753	0.734	0.812	0.730	0.710	0.744	0.700	0.711	0.732	0.722	0.710
Ho	0.816	0.800	0.812	0.730	0.770	0.744	0.700	0.711	0.732	0.782	0.769
He	0.869	0.871	0.896	0.863	0.854	0.854	0.825	0.835	0.833	0.829	0.845

The pairwise F_ST_ values between the sampled populations (areas) were low but after Bonferroni-correction still some significant differences (*p* < 0.0009) were found (highlighted in Table 2). This is especially the case for more distant geographical sampling areas, like North Sea and Balearic Sea, or North Sea and west coast of Sardinia. However, within the Mediterranean Sea some significant differences (Ionic Sea – west and east coast of Sardinia), were identified as well.

**Table 2:** F_ST_ values (below diagonal) and *p*-values (above diagonal) of pairwise comparison of the sampled areas regarding microsatellites; significant differentiations highlighted in bold: *p* = 0.05*; *p* = 0.0009** (Bonferroni-correction).

	North Sea	English Channel	Celtic Sea	Bay of Biscay	Gulf of Cadiz	Balearic Sea	West-Sardinia	East-Sardinia	Adriatic Sea	Ionian Sea	Aegean Sea
	(N)	(L)	(K)	(S)	C	(B)	(W)	(O)	(D)	(I)	(A)
North Sea (N)		0.541	0.9702	0.7293	0.4786	0	0	0.0026	0.1744	0	0.0131
English Channel (L)	0.001		0.171	0.3019	0.0553	0.0001	0.0017	0.0087	0.0272	0	0.0048
Celtic Sea (K)	0.005	0.0054		0.9035	0.1398	0.0022	0.0092	0.0012	0.0358	0.0038	0.0194
Bay of Biscay (S)	0.0005	0.0036	0.0038		0.2776	0.0048	0.0105	0.0057	0.0143	0.0238	0.0209
Gulf of Cadiz (C)	0.0011	0.0085	0.0074	0.0051		0.0041	0.0116	0.1727	0.5108	0.0079	0.1416
Balearic Sea (B)	0.0161**	0.0203**	0.0167*	0.0133*	0.0150*		0.0011	0	0.0013	0	0.0135
West-Sardinia (W)	0.0155**	0.0152*	0.0150*	0.0140*	0.0147*	0.0181*		0.2487	0.0013	0	0.0016
East-Sardinia (O)	0.0084*	0.0104*	0.0179*	0.0136*	0.0058	0.0255**	0.0041		0.0281	0.0003	0.0063
Adriatic Sea (D)	0.0046	0.0124*	0.0134*	0.0163*	0.0016	0.0223*	0.0240*	0.0133*		0.3277	0.6438
Ionian Sea (I)	0.0146**	0.0191**	0.0148*	0.0101*	0.0134*	0.0233**	0.0276**	0.0169**	0.0035		0.0212
Aegean Sea (A)	0.0087*	0.0144*	0.0140*	0.0135*	0.0076	0.0130*	0.0231*	0.0150*	0.001	0.0113*	

**Figure 2:**
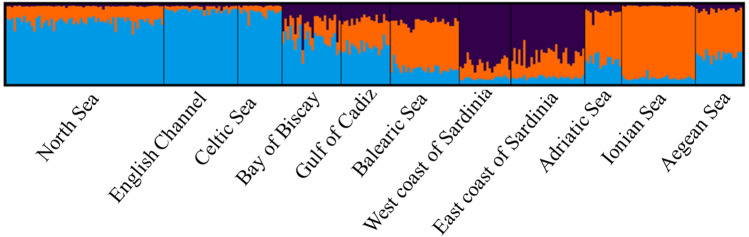
Structure analysis based on microsatellite data revealing three genetic clusters (blue = Atlantic cluster, orange & violet = Mediterranean cluster) for individuals from the sampled areas.

The STRUCTURE results revealed three clusters as the most likely number of clusters for this dataset (Fig. 2; Supplementary Fig. S2). One dominant cluster occurred in the Atlantic (Fig. 2; blue), while the Mediterranean Sea showed two different clusters (Fig. 2; violet and orange) with differences between the coasts of Sardinia and the remaining areas. The Bay of Biscay, the Gulf of Cadiz and the Balearic Sea represent a transition area with admixed origin from the three clusters in different proportions according to their respective geographic position. Whereas the Bay of Biscay is more genetically related to the East Atlantic samples (North Sea, English Channel and Celtic Sea), the Bay of Cadiz appears to represent equally the Atlantic and the Mediterranean, and the Balearic Sea is clearly more related to the Adriatic, Ionian and Aegean Seas.

### Phylogeography via COI-sequencing

**Figure 3:**
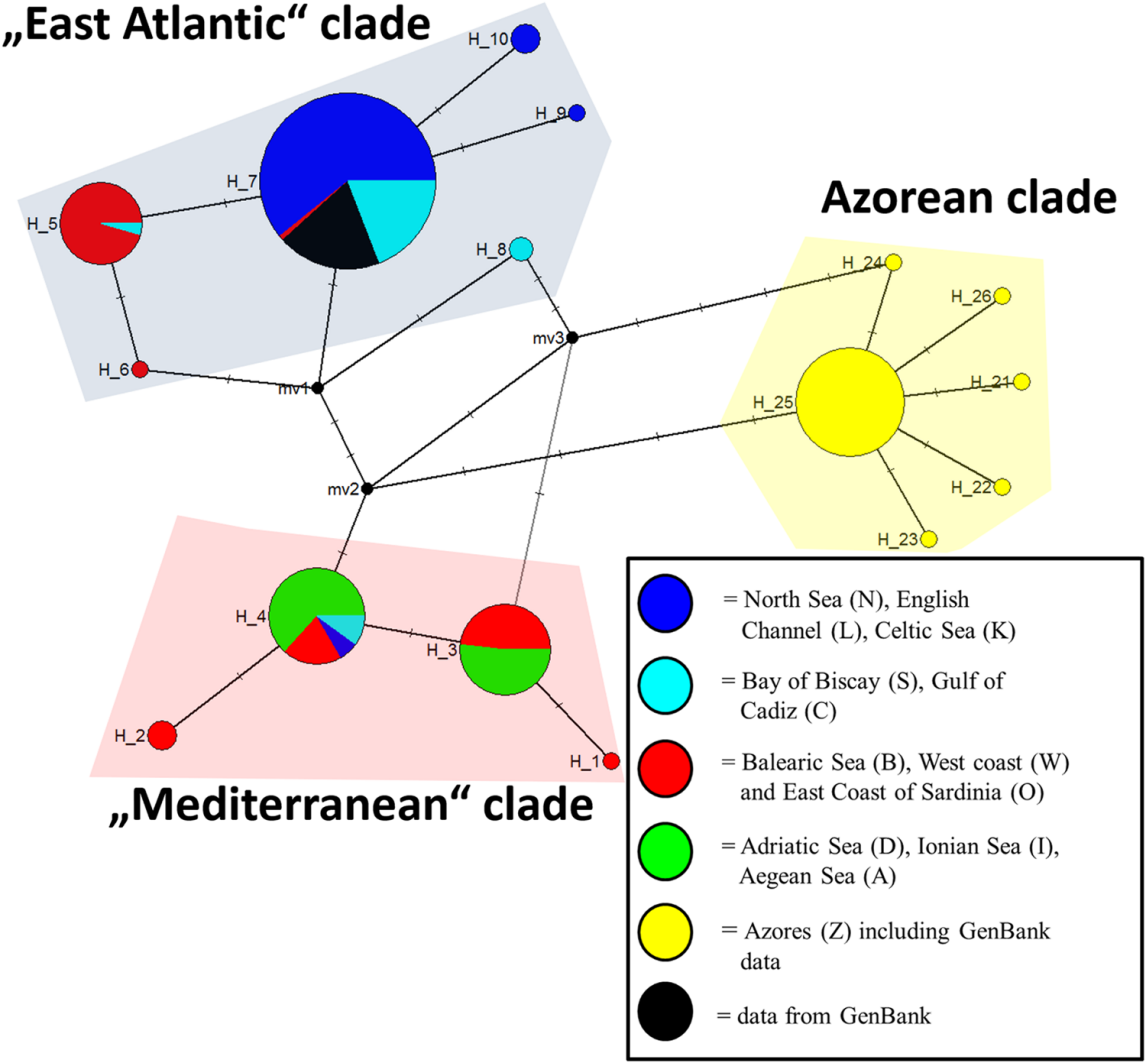
Haplotype network after Median-Joining Method for *L. forbesii* (haplotypes 1–10, 21–26) representing 249 COI-sequences (561 bp); black coloured GenBank data^33–37^ by various authors originate all from North East Atlantic individuals, yellow coloured including GenBank data^38–40^ .

The analysis of the mitochondrial COI-sequences identified 16 different haplotypes and illustrates the existence of two major clades, one exclusive of the Azores. The other clade can be split further into two clades, one representing mostly individuals from the East Atlantic, henceforth referred to as the “East Atlantic” clade, and the other representing mostly individuals from the Mediterranean, henceforth referred to as the “Mediterranean” clade (Fig. 3). All sequences were submitted to GenBank (Accession Numbers OK135754-OK135769).

**Figure 4:**
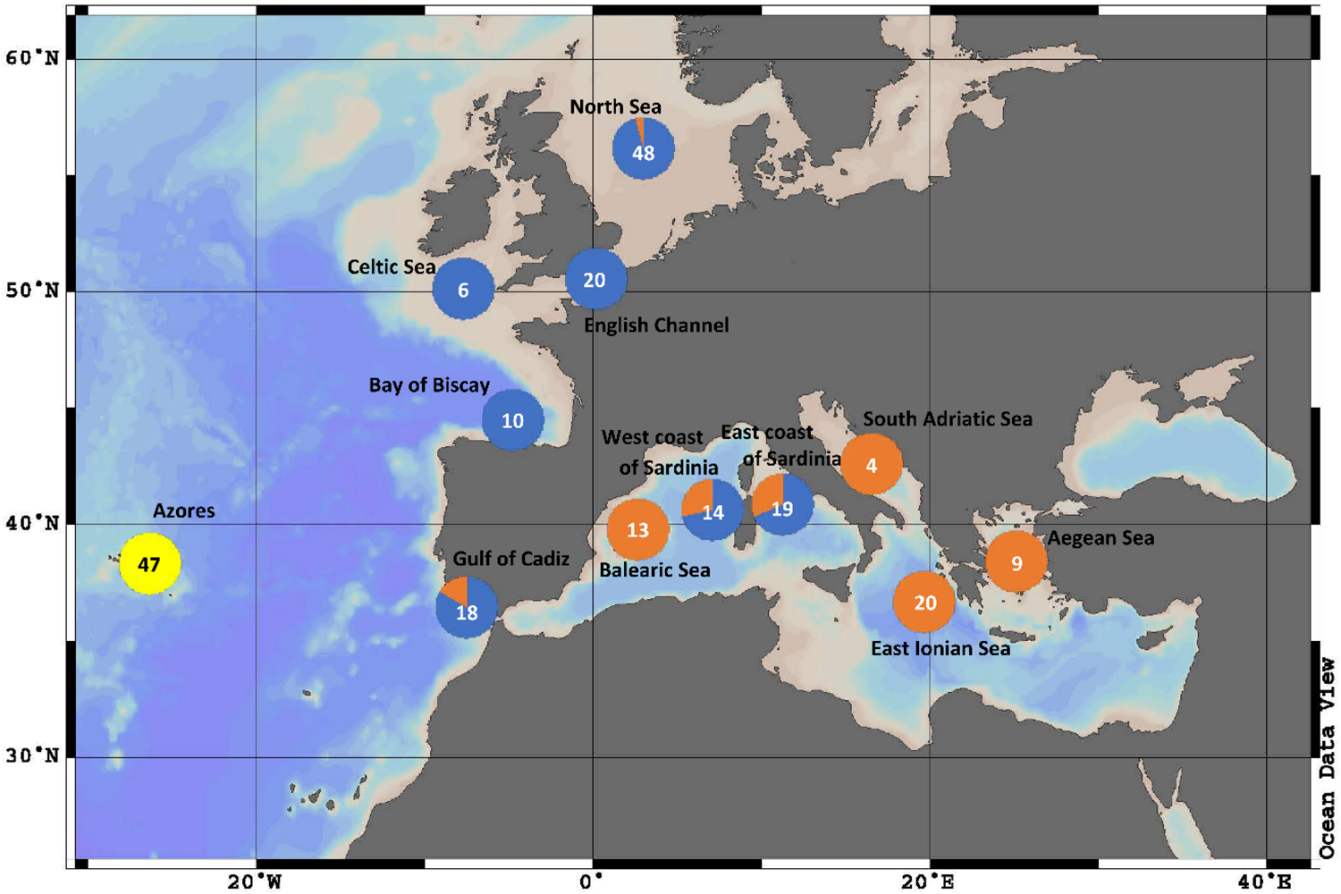
Geographical distribution of the Azores clade (yellow), “East Atlantic” clade (blue) and the “Mediterranean” clade (orange) of *L. forbesii* in Europe (sample size depicted inside circles) based on COI-sequences (Ocean Data View version 4.6.2).

The “East Atlantic” clade and “Mediterranean” clade were separated by at least three mutations, while the Azorean clade was separated by five and six mutations from the “Mediterranean” and the “East Atlantic” clades, respectively. Individuals caught in the eastern Mediterranean Sea (Aegean Sea, Ionic Sea, Adriatic Sea) belong exclusively to the “Mediterranean” clade, while individuals from the eastern Atlantic, with few exceptions, are members of the “East Atlantic” clade. The Gulf of Cadiz and the west and east coast of Sardinia represent a transition zone between the North Atlantic and the Mediterranean, with both clades occurring in these areas (Fig. 4).

The shorter and longer sequences show similar haplotype networks, although some mutations are omitted in the network performed with the shorter sequences length (see Supplementary Fig. S1, Table S5 online).

**Figure 5:**
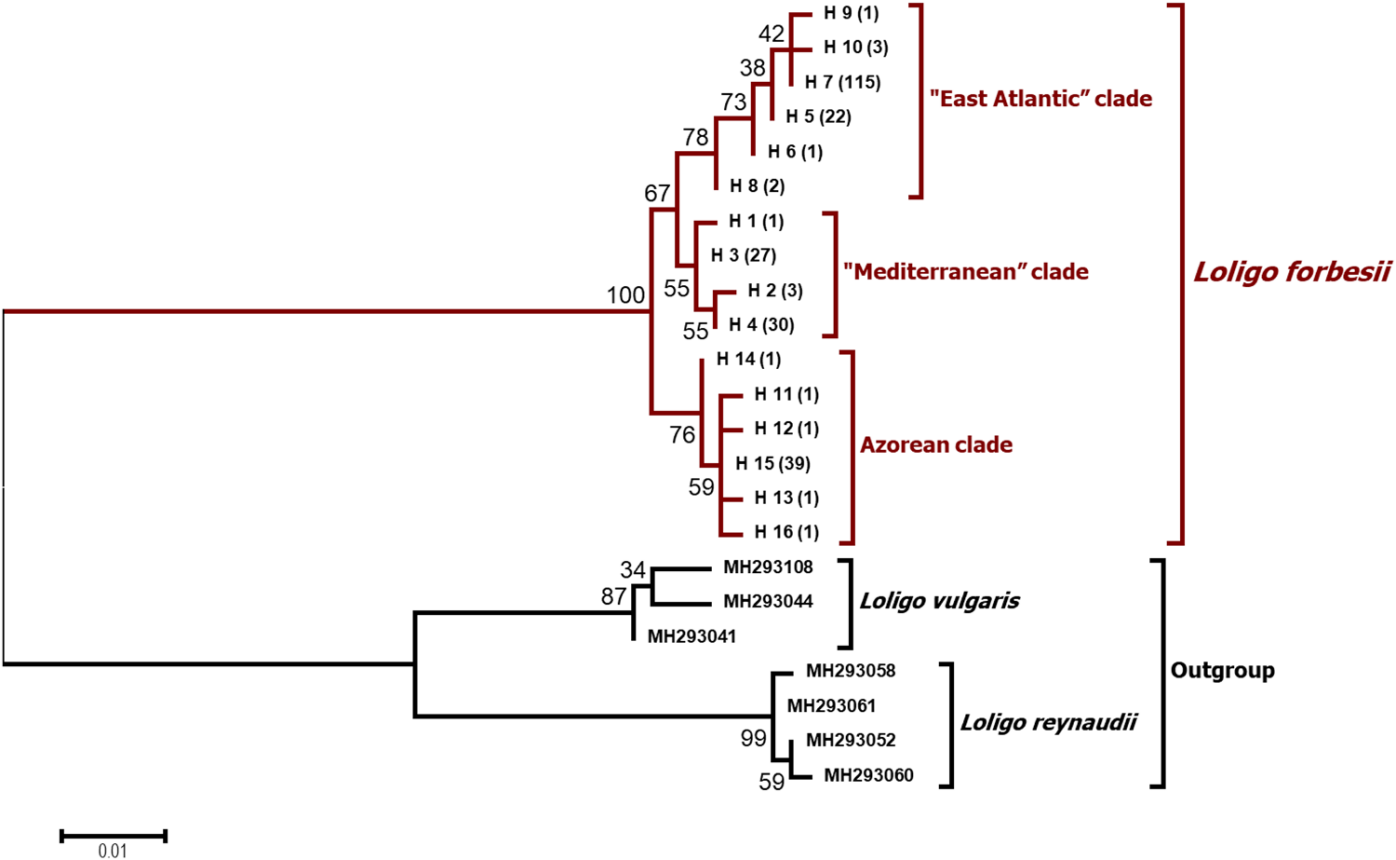
Molecular phylogenetic analysis after Maximum Likelihood (ML) method (1000 Bootstraps). Labelling next to the species names indicate the GenBank Accession Numbers for *L. reynaudii* and *L. vulgaris*.

The ML algorithm identify *L. forbesii* as a monophyletic species with 100% bootstrap support (Fig. 5). The sister group (outgroup) used consisting of *Loligo vulgaris* and *Loligo reynaudii* was identified in a more comprehensive analysis by calculating a ML tree with several loliginid species (Supplement Fig. S3). Within the species *L. forbesii,* two main lineages can be identified (Fig. 5). One lineage represents exclusively the individuals from the Azores and consists of the Azorean clade, whereas the other lineage consists of two more clades, the “East Atlantic” clade and the “Mediterranean” clade. The additional Bayesian phylogenetic analysis shows similar result with a comparable support of the clades (Supplement Fig. S4).

Based on clades defined by COI-sequence data, populations predominantly belonging to the same clade show low pairwise F_ST_ values based on microsatellites as well as relatively high gene flow rates (Table S5). For example, the North Sea individuals show high gene flow rates with other east Atlantic individuals like the fished individuals from the English Channel or the Celtic Sea (Nm = 16.49 and Nm = 12.97, respectively). Conversely, gene flow between the North Sea individuals and those from the eastern Mediterranean Sea, e.g. Ionic Sea and Aegean Sea, is far more restricted (Nm = 0.03 and Nm = 0.03, respectively; Table S5).

## Discussion

Our results based on *L. forbesii* microsatellites analysis illustrate the existence of three genetic units along the Atlantic and Mediterranean shelf area (North East Atlantic, western and eastern Mediterranean) with high gene flow between populations within and, partially, between the genetic clusters. Due to our substantially expanded sampling area and increased number of microsatellite loci in comparison to previous studies, we were able to identify a higher number of alleles giving us a more robust dataset for gene flow inferences^12,15,16,47,48^.

Locus Lfor7 showed up to eight peaks per individual, which was never described before^12,47^. This inconsistency between different studies may be due to different methods of allele scoring. Furthermore, loci Lfor14 and Lfor15 showed additional polymorphic amplification products. As unspecific priming and chain-termination can be excluded, we consider that these amplifications may eventually represent repeated regions in the genome. Not every locus delivers suitable results for every sample. This is not unusual as microsatellites are sensitive and the quality of the tissue sample plays an important role. Our dataset has 4.92% missing data, which is slightly higher than the 3.75% in Shaw and Boyle^47^. Not every locus was affected equally by missing data. The differences in the present study are most likely due to low DNA quality. This was especially the case for some samples from the Bay of Biscay and Adriatic Sea for which the ethanol may have not been changed adequately.

The observed low values of private allelic richness, as well as the low pairwise F_ST_ values are indicative of a considerable gene flow across the entire range of this species. Only sampling areas with a substantial geographic distance show low significant differences. Our STRUCTURE results show clear differences between Atlantic and Mediterranean *L. forbesii* individuals. The genetic composition of individuals from the Gulf of Cadiz to the western Mediterranean assigning to two or three genetic clusters is an evidence for recent gene flow, or otherwise a short period of genetic isolation. It is well known that the relatively shallow area of the Strait of Gibraltar has the potential to be a barrier for some species^49,50^. But it is not surprising that the area represents a transition zone for a shelf-living species such as *L. forbesii*. In addition, admixture within the Atlantic and the Mediterranean sampling sites seems to occur, represented by high gene flow rates (Supplementary Table S3), which is in line with the known migration behaviour of *L. forbesii*^2^. Other members of the taxon of Loliginidae also show gene flow over long geographic distances*.* Populations from *Doryteuthis opalescens* (formerly known as *Loligo opalescens*) and *Doryteuthis gahi* (formerly known as *Loligo gahi*) show poor genetic differentiation as well^51,52^. Our results from northern Atlantic sample sites correspond in general to the findings of Shaw et al.^12^ and Brierly et al.^11^. Hence, although we were not able to incorporate the Azorean samples in our microsatellite analysis, we anticipate that the Azores would represent a fourth microsatellite cluster as Shaw et al.^12^ uncovered significant genetic differences between *L. forbesii* from the archipelago and the North East Atlantic Shelf area.

We found a total of 16 COI-haplotypes for *L. forbesii*, whereof three have been reported before^32–40^ (H7 and H10, “East Atlantic” clade; H25, Azorean clade, see Fig. 3). We found three different mitochondrial clades in European waters: the exclusive Azorean clade, a clade dominated by East Atlantic individuals and a clade dominated by Mediterranean individuals. The two latter clades are not geographically isolated, because some individuals from the Atlantic and some individuals from the Mediterranean share some haplotypes from each clade. In detail, the “East Atlantic” clade is dominant in the Atlantic, while in the more eastern Mediterranean (Adriatic Sea, Ionian Sea, Aegean Sea) only members of the “Mediterranean” clade were found. Both clades co-occur in the Gulf of Cadiz as well as at the west and east coast of Sardinia, indicating an exchange of individuals between the Atlantic and Mediterranean Sea via the Strait of Gibraltar. Around the Balearic islands only members of the Mediterranean clade were found which may reflect the low number of samples. We found three North Sea individuals with Mediterranean haplotypes that were juvenile of 103–130 days old^53^ and were caught at a known spawning area during spawning time^54^. The elektropherogramms revealed that a sibling or half-sibling relationship can be excluded for these three individuals, additionally indicating multiple events of gene flow from the Mediterranean into the Atlantic. It is known that *Loligo reynaudii*, a closely related species, performs migrations over large geographical distances during spawning time and does not exhibit homing behavior^55^. When ready to spawn, individuals of this species show an average migration speed of 3 km per day, and exceptional migration events were also reported, as a movement of 207 km in 18 days^55^. While this information is not available for *L. forbesii*, it may be hypothesised that the spawning behaviour and the ability to move over long distances of *L. reynaudii* may be also present in *L. forbesii*.

The three mitochondrial clades could also be differentiated from the phylograms (Fig. 5, Supplement Fig. S3). The bootstrap values are rather low most likely reflecting the less than 1% overall differences for the COI-sequence data, but eventually also because the data was not partitioned by codon position. This low differentiation let us assume that the separation of the clades occurred in the Pleistocene^56^ (Supplement Fig. S5)*.* Our phylogeographic results are concordant with COI-data from *Sepia officinalis*, a species with a similar distribution to *L. forbesii*. Perez-Losada et al.^57^ inferred that *S. officinalis* expanded from Northwestern Europe coast into the Mediterranean, and observed that Aegean and Ionian populations of *S. officinalis* are genetically clearly separated from the remaining Mediterranean. We also found this differentiation between Eastern and Western Mediterranean but for *L. forbesii* mitochondrial haplotypes are generally more admixed between the sampling sites. This result may be due to gene flow following secondary contact between these clusters. Differences between local patterns of *L. forbesii* and *S. officinalis* may correspond to the different dispersal ability of the two species, with *L. forbesii* being a way more mobile species. Both the geological history of the Mediterranean and physical barriers between eastern and western Mediterranean Sea seem to have had a huge impact on the phylogeographical patterns of both cephalopod species.

The microsatellite and mitochondrial COI analyses show concurring results in population structure of *L. forbesii*. Comparing the same geographical range for both markers, we found two mitochondrial clades and three microsatellite clusters, which expectably express the different mutation rates of these markers. As microsatellites are known to better describe recent patterns of connectivity between populations^13^, our results reveal a recent differentiation into three clades within the European Shelf population.

Shaw et al.^12^ described the influence of geographic barriers, like deep sea basins, on the migration patterns of *L. forbesii*. The Italian peninsula as well as the Hellenic trench are potential migration barriers in the Mediterranean Sea. The offshore location of the Azores is very likely the reason for the genetic isolation between *L. forbesii* from the archipelago and the remaining North East Atlantic shelf area^12^, as the squids prefer the upper shelf slope^2,4^. Active migrations over large distances also seem to be important^58^ regarding the exchange between the Atlantic and Mediterranean over the Strait of Gibraltar. The two-layer water exchange in the Strait of Gibraltar described by Izquierdo et al.^59^ may influence the distribution of *L. forbesii*. Less dense Atlantic water forms a current in the upper water layer which flows into the Mediterranean, while the denser Mediterranean water forms the Mediterranean outflow, an underwater current which brings Mediterranean water masses into the Atlantic. This water exchange may affect differently adult *L. forbesii*, which spend the daytime close to the ground and ascend to surface waters at night for feeding^2^. This way, adults are directly influenced by the water flow in both directions, which supports the exchange of individuals between Atlantic and Mediterranean triggering the genetic admixture that can be seen in the present results. It is also expectable that the transport of paralarvae is influenced by currents.

With our new findings of the COI-gene analysis, we are able to describe uncovered phylogeographic patterns for *L. forbesii*. We found a subtle genetic differentiation between the Atlantic and the Mediterranean, but statoliths of Loligo, indistinguishable from those of current *L. forbesii* and *L. vulgaris*, were collected in early Miocene deposits at southern French shores^60^. This means that an ancestral of the current *L. forbesii* population existed in the Mediterranean sea, but we anticipate that the final establishment of the current Mediterranean population took place only after the Messinian Salinity Event 5.5 million years ago^61,62^, as the survival of the species during this crisis is very unlikely. We thus assume a repetitive colonialization of the Mediterranean by *L. forbesii* from the Atlantic across the Strait of Gibraltar. A geographical separation of the species took apparently place during the last ice age in the Late Pleistocene^63^, and the East Atlantic and Mediterranean lineage could evolve in allopatry. Today, the two previously evolved lineages have come into contact, supported by the combination of gene flow rates and the distribution of clade-specific haplotypes.

Under the assumption of the above mentioned scenario, the Sardinian cluster may have established following a second wave of immigration into the Mediterranean Sea. This cluster is represented in mitochondrial COI diversity by the “East Atlantic” haplotypes 5 and 6, which were only found around Sardinia. However, an alternative explanation for the genetic patterns observed is that a subpopulation of an ancestral population of *L. forbesii* that inhabited both the Atlantic and Mediterranean, got isolated in the Mediterranean due to sea level change eventually during the Late Pleistocene glaciations, becoming later the common ancestor of the three clades observed today, the “East Atlantic” clade, the “Mediterranean clade “ and the endemic clade in the Azores.

Based on our results, we recommend that future fishery management should consider at least three different genetic groups of *L. forbesii*. Even knowing that the structure analysis illustrates admixture in some regions, a more accurate classification into smaller management units seems not possible using the current genetic markers. Therefore, understanding the complex population structure of *L. forbesii* and differentiate stocks for a more precise management needs further research and more alternative methods. Some studies indicate that statolith shape analysis and statolith elemental analysis or the combination of both might be potential methods to identify smaller management units^64^.

## Supplementary Information

Supplementary Information.
