# Supplementary material for: Phylogeography of the veined squid, Loligo forbesii, in European waters
Source: Sci Rep. 2022 May 12;12:7817. doi: 10.1038/s41598-022-11530-z (PMC9098544; doi:10.1038/s41598-022-11530-z)
Supplement: Supplementary file 1 — Supplementary Information. [file 41598_2022_11530_MOESM1_ESM.docx]

**Phylogeography of the veined Squid, *Loligo forbesii,* in European Waters**

Anika Göpel^1,2^, Daniel Oesterwind^1*^, Christopher Barrett^3^, Rita Cannas^8^, Luis Silva Caparro^10^, Pierluigi Carbonara^9^, Marilena Donnaloia^9^, Maria Cristina Follesa^8^, Angela Larivain^6^, Vladimir Laptikhovsky^3^, Evgenia Lefkaditou^4^, Jean-Paul Robin^6^, Maria Begoña Santos^11^, Ignacio Sobrino^10^, Julio Valeiras^11^, Maria Valls^7^, Hugo C. Viera^12^, Kai Wieland^5,^ Ralf Bastrop^2^

^1^ Thünen Institute of Baltic Sea Fisheries, Alter Hafen Süd 2, 18069 Rostock, Germany

^2^ University of Rostock, Institute of Biological Sciences, Albert-Einstein-Str. 3, D-18059, Rostock, Germany

^3^ Cefas Laboratory, Pakefield Rd, Lowestoft NR33 0HT, UK

^4^ Hellenic Centre for Marine Research, Institute of Marine Biological Resources and Inland Waters, 576 SideRD Vouliagmenis Ave GR-16452, Athens, Greece

^5^ Technical University of Denmark, National Institute of Aquatic Resources, Nordsøen Forskerpark, Willemoesvej 2, DK-9850 Hirtshals, Denmark

^6^ University of Caen Normandy, CS 14032, 14032, Caen cedex 05, France

^7^ Instituto Español de Oceanografía (IEO), Centre Oceanográfic de les Balears s/n, 07015 Palma, Spain

^8^ Department of Life and Environmental Sciences, University of Cagliari, Cagliari, Italy

^9^ COISPA Tecnologia & Ricerca, via dei Trulli, 18-20, Bari, Italy.

^10^ Instituto Español de Oceanografía, Centro Oceanográfico de Cádiz, Puerto Pesquero, Muelle de Levante s/n, 11006 Cádiz, Spain

^11^ Instituto Español de Oceanografía (IEO), Centro Oceanográfico de Vigo, Subida a Radio Faro, 50, 36390 Vigo, Spain

^12^ CESAM ‑ Centre for Environmental and Marine Studies, Department of Biology, University of Aveiro, Campus de Santiago, 3810‑193 Aveiro, Portugal

*corresponding Author: daniel.oesterwind@thuenen.de

**Supplementary Tables and Figures**

**Table S1.** Number of individuals per sampled areas.

| **Sampled area** | **Number of individuals** |
| --- | --- |
| Aegean Sea (A) | 19 |
| Balearic Sea (B) | 28 |
| Gulf of Cadiz (C) | 20 |
| South Adriatic Sea (D) | 15 |
| East Ionian Sea (I) | 30 |
| Celtic Sea (K) | 19 |
| English Channel (L) | 30 |
| North Sea (N) | 64 |
| East coast of Sardinia (O) | 30 |
| Bay of Biscay (S) | 24 |
| West coast of Sardinia (W) | 21 |
| Azores (Z) | 47 |

**Table S2.** Internal primers for the COI-gene.

| Forward primer | Reverse primer |
| --- | --- |
| GCTGTAGAAAGAGGGGCTGGTAC | GGAGCAATTAACTTCATCACAACC |
| AGGGCCTTCAGTAGATTTAGCC | GGAGCAATTACAATACTTTTAACTGAC |

**Table S3**. Gene flow (Nm; below the diagonale) and F_ST_ value (above the diagonale) for COI-data; Data from Adriatic Sea (D) not included due to low number of sequences.

|  | North Sea (N) | English Channel (L) | Celtic Sea (K) | Bay of Biscay (S) | Gulf of Cadiz (C) | Balearic Sea (B) | West coast of Sardinia (W) | East coast of Sardinia (O) | East Ionian Sea (I) | Aegean Sea (A) | Azores (Z) |
| --- | --- | --- | --- | --- | --- | --- | --- | --- | --- | --- | --- |
| Total (N) | 48 | 20 | 6 | 10 | 18 | 13 | 14 | 19 | 20 | 9 | 41 |
| N |  | 0.015 | 0.019 | 0.047 | 0.033 | 0.947 | 0.290 | 0.333 | 0.899 | 0.904 | 0.957 |
| L | 16.49 |  | 0.000 | 0.081 | 0.109 | 0.975 | 0.343 | 0.934 | 0.934 | 0.936 | 0.976 |
| K | 12.97 | n.d. |  | 0.089 | 0.118 | 0.985 | 0.352 | 0.396 | 0.945 | 0.946 | 0.983 |
| S | 5.10 | 2.84 | 2.56 |  | 0.051 | 0.888 | 0.204 | 0.260 | 0.839 | 0.843 | 0.920 |
| C | 7.31 | 2.03 | 1.88 | 4.64 |  | 0.849 | 0.167 | 0.775 | 0.775 | 0.790 | 0.895 |
| B | 0.01 | 0.01 | 0.00 | 0.03 | 0.04 |  | 0.695 | 0.714 | 0.565 | 0.203 | 0.968 |
| W | 0.61 | 0.48 | 0.46 | 0.98 | 1.25 | 0.11 |  | -0.061 | 0.603 | 0.627 | 0.804 |
| O | 0.50 | 0.40 | 0.38 | 0.71 | 0.97 | 0.10 | -4.36 |  | 0.618 | 0.645 | 0.813 |
| I | 0.03 | 0.02 | 0.01 | 0.05 | 0.07 | 0.19 | 0.16 | 0.15 |  | 0.110 | 0.934 |
| A | 0.03 | 0.02 | 0.01 | 0.05 | 0.07 | 0.98 | 0.15 | 0.14 | 2.02 |  | 0.935 |
| Z | 0.01 | 0.01 | 0.00 | 0.02 | 0.03 | 0.01 | 0.06 | 0.06 | 0.02 | 0.01 |  |

**Table S4.** Overview of the values of the microsatellite analysis; allelic richness and private allelic richness (pAR) based on 15 individuals; Ho = observed heterozygosity; He = expected Heterozygosity; significant deviations marked with *.

|  | North Sea | English Channel | Celtic Sea | Bay of Biscay | Gulf of Cadiz | Balearic Sea | West-Sardinia | East-Sardinia | Adriatic Sea | Ionian Sea | Aegean Sea |
| --- | --- | --- | --- | --- | --- | --- | --- | --- | --- | --- | --- |
| n | 64 | 30 | 19 | 24 | 20 | 28 | 21 | 30 | 15 | 30 | 19 |
| Lfor1 | **total allele number: 20** | | |  |  |  |  |  |  |  |  |
| allele number | 17 | 11 | 13 | 12 | 10 | 10 | 11 | 12 | 11 | 11 | 8 |
| allelic richness | 11.236 | 8.831 | 12.279 | 10.329 | 9.476 | 7.797 | 9.824 | 9.838 | 11.000 | 7.221 | 6.461 |
| fragment size | 137-188 | 137-182 | 128-170 | 137-170 | 137-182 | 137-182 | 137-173 | 137-179 | 137-188 | 137-182 | 137-170 |
| pAR | 0.247 | 0.033 | 1.667 | 0.000 | 0.044 | 0.010 | 0.000 | 0.002 | 0.994 | 0.010 | 0.000 |
| Ho | 0.475* | 0.400* | 0.778 | 0.333* | 0.500* | 0.393* | 0.450* | 0.357* | 0.308* | 0.357* | 0.444* |
| He | 0.892* | 0.850* | 0.927 | 0.887* | 0.894* | 0.798* | 0.845* | 0.882* | 0.911* | 0.858* | 0.803* |
| Lfor2 | **total allele number: 11** | | |  |  |  |  |  |  |  |  |
| allele number | 10 | 11 | 9 | 8 | 9 | 9 | 7 | 8 | 8 | 8 | 8 |
| allelic richness | 7.752 | 9.260 | 8.508 | 7.286 | 8.176 | 7.885 | 6.815 | 7.065 | 8.000 | 6.687 | 7.322 |
| fragment size | 133-160 | 133-163 | 133-157 | 133-163 | 133-160 | 133-160 | 133-157 | 133-157 | 133-154 | 133-160 | 133-160 |
| pAR | 0.000 | 0.040 | 0.000 | 0.066 | 0.000 | 0.000 | 0.000 | 0.000 | 0.001 | 0.000 | 0.000 |
| Ho | 0.810* | 0.833 | 0.765 | 0.792 | 0.950 | 0.786 | 0.800 | 0.767 | 0.667 | 0.800 | 0.789 |
| He | 0.793* | 0.846 | 0.761 | 0.771 | 0.799 | 0.797 | 0.794 | 0.777 | 0.694 | 0.723 | 0.750 |
| Lfor3 | **total allele number:32** | | |  |  |  |  |  |  |  |  |
| allele number | 22 | 17 | 19 | 16 | 15 | 16 | 13 | 19 | 10 | 16 | 14 |
| allelic richness | 15.426 | 13.702 | 17.531 | 13.338 | 14.185 | 12.615 | 11.479 | 15.042 | 10.000 | 11.896 | 12.642 |
| fragment size | 102-192 | 108-192 | 102-174 | 108-177 | 105-165 | 111-171 | 108-183 | 108-195 | 108-168 | 90-168 | 108-180 |
| pAR | 0.282 | 0.514 | 0.368 | 0.233 | 0.213 | 0.012 | 0.546 | 0.848 | 0.053 | 0.678 | 0.791 |
| Ho | 0.930 | 0.900 | 1.000 | 0.870 | 0.842 | 0.893 | 1.000 | 0.815 | 0.667 | 0.767* | 0.789 |
| He | 0.938 | 0.932 | 0.963 | 0.925 | 0.916 | 0.909 | 0.911 | 0.936 | 0.841 | 0.849* | 0.906 |
| Lfor4 | **total allele number: 12** | | |  |  |  |  |  |  |  |  |
| allele number | 10 | 9 | 9 | 9 | 8 | 10 | 7 | 7 | 8 | 11 | 9 |
| allelic richness | 8.161 | 8.124 | 8.743 | 8.049 | 7.665 | 8.215 | 6.633 | 6.401 | 8.000 | 8.999 | 8.492 |
| fragment size | 202-229 | 202-229 | 202-232 | 202-226 | 202-229 | 202-235 | 202-229 | 202-223 | 205-229 | 202-235 | 202-232 |
| pAR | 0.002 | 0.000 | 0.093 | 0.003 | 0.000 | 0.056 | 0.000 | 0.000 | 0.000 | 0.066 | 0.086 |
| Ho | 0.922 | 0.900 | 0.824 | 0.833 | 0.800 | 0.821 | 0.810 | 0.828 | 0.692 | 0.933 | 0.368 |
| He | 0.832 | 0.839 | 0.863 | 0.828 | 0.828 | 0.755 | 0.819 | 0.822 | 0.812 | 0.838 | 0.839 |
| Lfor5 | **total allele number: 27** | | |  |  |  |  |  |  |  |  |
| allele number | 17 | 17 | 17 | 11 | 11 | 18 | 9 | 12 | 12 | 14 | 10 |
| allelic richness | 11.476 | 12.397 | 15.465 | 9.418 | 10.324 | 13.036 | 8.057 | 10.024 | 12.000 | 10.721 | 9.615 |
| fragment size | 97-147 | 79-129 | 97-163 | 95-119 | 97-123 | 95-131 | 97-117 | 97-127 | 97-129 | 97-133 | 97-121 |
| pAR | 0.594 | 0.978 | 4.413 | 0.160 | 0.004 | 0.576 | 0.003 | 0.009 | 0.024 | 0.084 | 0.366 |
| Ho | 0.797 | 0.867 | 0.944 | 0.875 | 0.850 | 0.893 | 0.714 | 0.733 | 0.867 | 0.800 | 0.889 |
| He | 0.876 | 0.898 | 0.944 | 0.866 | 0.859 | 0.914 | 0.804 | 0.867 | 0.917 | 0.856 | 0.867 |
| Lfor6 | **total allele number: 24** | | |  |  |  |  |  |  |  |  |
| allele number | 15 | 14 | 14 | 14 | 9 | 12 | 9 | 8 | 7 | 9 | 10 |
| allelic richness | 9.523 | 9.872 | 13.063 | 11.317 | 7.635 | 9.420 | 7.267 | 6.957 | 7.000 | 7.337 | 9.037 |
| fragment size | 86-126 | 84-122 | 78-120 | 82-150 | 82-124 | 82-126 | 86-118 | 86-110 | 86-116 | 82-122 | 86-118 |
| pAR | 0.094 | 0.042 | 2.430 | 1.267 | 0.834 | 0.171 | 0.000 | 0.000 | 0.000 | 0.065 | 0.961 |
| Ho | 0.734 | 0.600 | 0.667* | 0.708 | 0.500 | 0.786 | 0.571 | 0.533 | 0.643 | 0.600 | 0.737 |
| He | 0.740 | 0.746 | 0.898* | 0.808 | 0.594 | 0.783 | 0.595 | 0.605 | 0.651 | 0.623 | 0.708 |
| Lfor8 | **total allele number: 30** | | |  |  |  |  |  |  |  |  |
| allele number | 25 | 18 | 13 | 8 | 13 | 11 | 6 | 11 | 9 | 13 | 12 |
| allelic richness | 13.216 | 14.125 | 13.000 | 7.267 | 11.134 | 9.581 | 6.000 | 9.414 | 9.000 | 10.460 | 11.116 |
| fragment size | 96-276 | 180-264 | 96-261 | 189-210 | 96-249 | 189-270 | 192-252 | 186-261 | 189-216 | 96-258 | 192-261 |
| pAR | 0.938 | 1.488 | 0.257 | 0.000 | 0.057 | 0.751 | 0.000 | 0.538 | 1.004 | 0.023 | 0.099 |
| Ho | 0.823 | 0.643* | 0.750 | 0.826 | 0.700 | 0.440* | 0.267* | 0.524 | 0.750 | 0.931 | 0.722 |
| He | 0.906 | 0.897* | 0.902 | 0.829 | 0.868 | 0.886* | 0.754* | 0.830 | 0.867 | 0.878 | 0.892 |
| Lfor10 | **total allele number: 21** | | |  |  |  |  |  |  |  |  |
| allele number | 14 | 10 | 12 | 10 | 9 | 10 | 9 | 9 | 9 | 9 | 12 |
| allelic richness | 8.694 | 7.949 | 11.496 | 8.792 | 8.822 | 8.804 | 8.666 | 7.664 | 9.000 | 7.773 | 10.933 |
| fragment size | 111-198 | 96-189 | 162-561 | 108-195 | 117-192 | 108-192 | 108-189 | 117-195 | 96-195 | 168-195 | 117-198 |
| pAR | 0.507 | 0.019 | 3.440 | 0.036 | 0.001 | 0.024 | 0.059 | 0.001 | 0.501 | 0.000 | 0.128 |
| Ho | 0.891 | 0.900 | 0.647* | 0.870 | 0.800 | 0.889 | 0.750 | 0.862 | 1.000 | 0.867 | 0.842 |
| He | 0.860 | 0.824 | 0.891* | 0.861 | 0.886 | 0.868 | 0.883 | 0.781 | 0.880 | 0.836 | 0.900 |
| Lfor11 | **total allele number: 24** | | |  |  |  |  |  |  |  |  |
| allele number | 17 | 17 | 12 |  | 15 | 14 | 14 | 13 |  | 13 | 15 |
| allelic richness | 12.771 | 13.834 | 12.000 |  | 13.581 | 11.614 | 13.378 | 11.295 |  | 10.974 | 14.358 |
| fragment size | 188-242 | 182-239 | 182-242 |  | 197-245 | 191-248 | 197-242 | 197-239 |  | 189-242 | 191-251 |
| pAR | 0.000 | 0.000 | 0.000 |  | 0.411 | 0.000 | 0.000 | 0.000 |  | 0.000 | 0.000 |
| Ho | 0.919 | 0.897* | 0.923 |  | 0.900 | 0.885 | 0.706 | 0.750 |  | 0.800 | 0.706 |
| He | 0.922 | 0.936* | 0.929 |  | 0.935 | 0.902 | 0.932 | 0.880 |  | 0.890 | 0.943 |
| Lfor12 | **total allele number: 49** | | |  |  |  |  |  |  |  |  |
| allele number | 43 | 31 | 21 | 26 | 24 | 22 | 17 | 19 | 13 | 17 | 18 |
| allelic richness | 21.378 | 21.662 | 21.000 | 20.323 | 19.944 | 16.001 | 14.788 | 15.081 | 13.000 | 13.438 | 16.783 |
| fragment size | 178-322 | 193-322 | 211-322 | 196-319 | 181-316 | 181-298 | 199-313 | 184-283 | 202-259 | 205-313 | 202-310 |
| pAR | 1.042 | 0.463 | 0.878 | 0.592 | 0.519 | 0.493 | 0.097 | 0.440 | 0.005 | 0.033 | 0.013 |
| Ho | 0.844* | 1.000 | 0.923 | 0.696* | 0.737* | 0.714 | 0.900 | 0.769 | 0.692 | 0.900 | 0.889 |
| He | 0.975* | 0.977 | 0.982 | 0.968* | 0.967* | 0.944 | 0.928 | 0.938 | 0.923 | 0.928 | 0.956 |
| Lfor13 | **total allele number: 16** | | |  |  |  |  |  |  |  |  |
| allele number | 11 | 12 | 10 | 10 | 10 | 8 | 10 | 10 | 7 | 9 | 10 |
| allelic richness | 8.728 | 9.575 | 10.000 | 9.294 | 9.180 | 7.326 | 9.589 | 9.294 | 7.000 | 7.630 | 9.426 |
| fragment size | 229-253 | 217-253 | 229-253 | 225-243 | 229-247 | 227-243 | 227-247 | 227-255 | 229-243 | 231-249 | 229-249 |
| pAR | 0.000 | 0.559 | 0.287 | 0.715 | 0.000 | 0.000 | 0.001 | 0.715 | 0.000 | 0.042 | 0.194 |
| Ho | 0.813 | 0.897 | 0.750 | 0.476* | 0.900 | 0.577 | 0.667 | 0.667 | 0.833 | 0.793 | 0.944 |
| He | 0.830 | 0.861 | 0.859 | 0.880* | 0.882 | 0.817 | 0.822 | 0.882 | 0.879 | 0.815 | 0.856 |
| Lfor16 | **total allele number: 16** | | |  |  |  |  |  |  |  |  |
| allele number | 14 | 10 | 9 | 12 | 7 | 11 | 7 | 12 | 9 | 10 | 8 |
| allelic richness | 9.184 | 8.373 | 9.000 | 9.847 | 6.723 | 9.710 | 6.409 | 9.884 | 9.000 | 8.464 | 7.662 |
| fragment size | 138-164 | 142-160 | 140-160 | 136-162 | 142-156 | 138-162 | 142-156 | 140-164 | 142-164 | 140-160 | 140-166 |
| pAR | 0.011 | 0.000 | 0.020 | 0.629 | 0.000 | 0.140 | 0.000 | 0.003 | 0.011 | 0.000 | 0.790 |
| Ho | 0.828 | 0.700 | 0.769 | 0.750 | 0.750 | 0.857 | 0.762 | 0.923 | 0.929 | 0.833 | 0.789 |
| He | 0.839 | 0.835 | 0.834 | 0.871 | 0.817 | 0.870 | 0.815 | 0.824 | 0.786 | 0.850 | 0.725 |

**Table S5.** Overview of all investigated isolates and GenBank samples with geographic origin and haplotype in both networks. The short sequences correspond to the long sequences from bp63 to bp494. Unless otherwise stated, all samples were collected by the authors themselves.

| **Isolate number/**  **Accession number** | **geographic origin** | **haplotype in long network*** | **haplotype in short network** | **Source** |
| --- | --- | --- | --- | --- |
| **A1** | Aegean Sea |  | H14 | This study |
| **A2** | Aegean Sea | H3 | H14 | This study |
| **A3** | Aegean Sea |  |  | This study |
| **A4** | Aegean Sea | H3 | H14 | This study |
| **A5** | Aegean Sea |  |  | This study |
| **A6** | Aegean Sea | H3 | H14 | This study |
| **A7** | Aegean Sea | H3 | H14 | This study |
| **A8** | Aegean Sea | H4 | H14 | This study |
| **A9** | Aegean Sea |  |  | This study |
| **A10** | Aegean Sea |  | H14 | This study |
| **A11** | Aegean Sea |  | H14 | This study |
| **A12** | Aegean Sea |  |  | This study |
| **A13** | Aegean Sea |  |  | This study |
| **A14** | Aegean Sea | H3 | H14 | This study |
| **A15** | Aegean Sea | H4 | H14 | This study |
| **A16** | Aegean Sea | H3 | H14 | This study |
| **A17** | Aegean Sea | H4 | H14 | This study |
| **A18** | Aegean Sea |  | H14 | This study |
| **A19** | Aegean Sea |  | H14 | This study |
| **B1** | Balearic Sea |  | H14 | This study |
| **B2** | Balearic Sea |  |  | This study |
| **B3** | Balearic Sea |  | H14 | This study |
| **B4** | Balearic Sea |  |  | This study |
| **B5** | Balearic Sea | H3 | H14 | This study |
| **B6** | Balearic Sea |  | H14 | This study |
| **B7** | Balearic Sea |  | H14 | This study |
| **B8** | Balearic Sea |  | H13 | This study |
| **B9** | Balearic Sea | H3 | H14 | This study |
| **B10** | Balearic Sea |  |  | This study |
| **B11** | Balearic Sea |  | H14 | This study |
| **B12** | Balearic Sea |  | H14 | This study |
| **B13** | Balearic Sea | H3 | H14 | This study |
| **B14** | Balearic Sea | H3 | H14 | This study |
| **B15** | Balearic Sea |  |  | This study |
| **B16** | Balearic Sea | H3 | H14 | This study |
| **B17** | Balearic Sea |  | H14 | This study |
| **B18** | Balearic Sea | H1 | H11 | This study |
| **B19** | Balearic Sea | H3 | H14 | This study |
| **B20** | Balearic Sea |  |  | This study |
| **B21** | Balearic Sea | H3 | H14 | This study |
| **B22** | Balearic Sea | H3 | H14 | This study |
| **B23** | Balearic Sea |  |  | This study |
| **B24** | Balearic Sea | H3 | H14 | This study |
| **B25** | Balearic Sea |  |  | This study |
| **B26** | Balearic Sea | H3 | H14 | This study |
| **B27** | Balearic Sea | H3 | H14 | This study |
| **B28** | Balearic Sea | H3 | H14 | This study |
| **C1** | Gulf of Cadiz | H7 | H17 | This study |
| **C2** | Gulf of Cadiz | H7 | H17 | This study |
| **C3** | Gulf of Cadiz | H7 | H17 | This study |
| **C4** | Gulf of Cadiz |  | H17 | This study |
| **C5** | Gulf of Cadiz | H7 | H17 | This study |
| **C6** | Gulf of Cadiz | H7 | H17 | This study |
| **C7** | Gulf of Cadiz | H4 | H14 | This study |
| **C8** | Gulf of Cadiz | H7 | H17 | This study |
| **C9** | Gulf of Cadiz | H4 | H14 | This study |
| **C10** | Gulf of Cadiz | H7 | H17 | This study |
| **C11** | Gulf of Cadiz | H7 | H17 | This study |
| **C12** | Gulf of Cadiz | H7 | H17 | This study |
| **C13** | Gulf of Cadiz | H7 | H17 | This study |
| **C14** | Gulf of Cadiz |  | H17 | This study |
| **C15** | Gulf of Cadiz | H7 | H17 | This study |
| **C16** | Gulf of Cadiz | H7 | H17 | This study |
| **C17** | Gulf of Cadiz | H7 | H17 | This study |
| **C18** | Gulf of Cadiz | H4 | H14 | This study |
| **C19** | Gulf of Cadiz | H7 | H17 | This study |
| **C20** | Gulf of Cadiz | H7 | H17 | This study |
| **D1** | South Adriatic Sea |  |  | This study |
| **D2** | South Adriatic Sea |  |  | This study |
| **D3** | South Adriatic Sea | H4 | H14 | This study |
| **D4** | South Adriatic Sea |  |  | This study |
| **D5** | South Adriatic Sea | H4 | H14 | This study |
| **D6** | South Adriatic Sea | H3 | H14 | This study |
| **D7** | South Adriatic Sea | H4 | H14 | This study |
| **D8** | South Adriatic Sea |  |  | This study |
| **D9** | South Adriatic Sea |  |  | This study |
| **D10** | South Adriatic Sea |  |  | This study |
| **D11** | South Adriatic Sea |  |  | This study |
| **D12** | South Adriatic Sea |  |  | This study |
| **D13** | South Adriatic Sea |  |  | This study |
| **D14** | South Adriatic Sea |  |  | This study |
| **D15** | South Adriatic Sea |  |  | This study |
| **D16** | South Adriatic Sea |  |  | This study |
| **D17** | South Adriatic Sea |  |  | This study |
| **D18** | South Adriatic Sea |  |  | This study |
| **I1** | East Ionian Sea |  | H14 | This study |
| **I2** | East Ionian Sea | H4 | H14 | This study |
| **I3** | East Ionian Sea | H3 | H14 | This study |
| **I4** | East Ionian Sea | H4 | H14 | This study |
| **I5** | East Ionian Sea | H3 | H14 | This study |
| **I6** | East Ionian Sea | H3 | H14 | This study |
| **I7** | East Ionian Sea | H4 | H14 | This study |
| **I8** | East Ionian Sea |  | H14 | This study |
| **I9** | East Ionian Sea | H3 | H14 | This study |
| **I10** | East Ionian Sea |  | H14 | This study |
| **I11** | East Ionian Sea |  | H14 | This study |
| **I12** | East Ionian Sea |  | H14 | This study |
| **I13** | East Ionian Sea |  | H14 | This study |
| **I14** | East Ionian Sea | H3 | H14 | This study |
| **I15** | East Ionian Sea | H4 | H14 | This study |
| **I16** | East Ionian Sea | H4 | H14 | This study |
| **I17** | East Ionian Sea | H4 | H14 | This study |
| **I18** | East Ionian Sea |  | H14 | This study |
| **I19** | East Ionian Sea | H4 | H14 | This study |
| **I20** | East Ionian Sea |  | H14 | This study |
| **I21** | East Ionian Sea |  | H14 | This study |
| **I22** | East Ionian Sea |  | H14 | This study |
| **I23** | East Ionian Sea | H4 | H14 | This study |
| **I24** | East Ionian Sea | H3 | H14 | This study |
| **I25** | East Ionian Sea | H4 | H14 | This study |
| **I26** | East Ionian Sea | H4 | H14 | This study |
| **I27** | East Ionian Sea | H3 | H14 | This study |
| **I28** | East Ionian Sea | H4 | H14 | This study |
| **I29** | East Ionian Sea | H4 | H14 | This study |
| **I30** | East Ionian Sea | H4 | H14 | This study |
| **K1** | Celtic Sea |  |  | This study |
| **K2** | Celtic Sea | H7 | H17 | This study |
| **K3** | Celtic Sea |  |  | This study |
| **K4** | Celtic Sea | H7 | H17 | This study |
| **K5** | Celtic Sea | H7 | H17 | This study |
| **K6** | Celtic Sea |  |  | This study |
| **K7** | Celtic Sea |  |  | This study |
| **K8** | Celtic Sea | H7 | H17 | This study |
| **K9** | Celtic Sea |  |  | This study |
| **K10** | Celtic Sea |  | H17 | This study |
| **K11** | Celtic Sea |  |  | This study |
| **K12** | Celtic Sea |  | H17 | This study |
| **K13** | Celtic Sea |  |  | This study |
| **K14** | Celtic Sea | H7 | H17 | This study |
| **K15** | Celtic Sea |  |  | This study |
| **K16** | Celtic Sea | H7 | H17 | This study |
| **K17** | Celtic Sea |  | H17 | This study |
| **K18** | Celtic Sea |  | H17 | This study |
| **K19** | Celtic Sea |  |  | This study |
| **L1** | English Channel | H7 | H17 | This study |
| **L2** | English Channel | H7 | H17 | This study |
| **L3** | English Channel | H7 | H17 | This study |
| **L4** | English Channel | H7 | H17 | This study |
| **L5** | English Channel | H7 | H17 | This study |
| **L6** | English Channel |  |  | This study |
| **L7** | English Channel |  | H17 | This study |
| **L8** | English Channel | H7 | H17 | This study |
| **L9** | English Channel | H7 | H17 | This study |
| **L10** | English Channel | H9 | H17 | This study |
| **L11** | English Channel |  | H17 | This study |
| **L12** | English Channel | H7 | H17 | This study |
| **L13** | English Channel | H7 | H17 | This study |
| **L14** | English Channel |  |  | This study |
| **L15** | English Channel |  | H17 | This study |
| **L16** | English Channel | H7 | H17 | This study |
| **L17** | English Channel | H7 | H17 | This study |
| **L18** | English Channel |  |  | This study |
| **L19** | English Channel |  |  | This study |
| **L20** | English Channel | H7 | H17 | This study |
| **L21** | English Channel | H7 | H17 | This study |
| **L22** | English Channel | H7 | H17 | This study |
| **L23** | English Channel |  | H17 | This study |
| **L24** | English Channel | H7 | H17 | This study |
| **L25** | English Channel | H7 | H17 | This study |
| **L26** | English Channel | H7 | H17 | This study |
| **L27** | English Channel | H7 | H17 | This study |
| **L28** | English Channel |  |  | This study |
| **L29** | English Channel |  |  | This study |
| **L30** | English Channel | H7 | H17 | This study |
| **N26** | North Sea |  |  | This study |
| **N27** | North Sea |  | H17 | This study |
| **N29** | North Sea | H7 | H17 | This study |
| **N30** | North Sea |  |  | This study |
| **N31** | North Sea | H7 | H17 | This study |
| **N32** | North Sea | H7 | H17 | This study |
| **N33** | North Sea | H7 | H17 | This study |
| **N34** | North Sea | H7 | H17 | This study |
| **N35** | North Sea | H7 | H17 | This study |
| **N36** | North Sea | H7 | H17 | This study |
| **N37** | North Sea | H7 | H17 | This study |
| **N38** | North Sea | H7 | H17 | This study |
| **N39** | North Sea | H7 | H17 | This study |
| **N40** | North Sea | H7 | H17 | This study |
| **N41** | North Sea | H7 | H17 | This study |
| **N42** | North Sea | H7 | H17 | This study |
| **N43** | North Sea | H7 | H17 | This study |
| **N44** | North Sea | H7 | H17 | This study |
| **N45** | North Sea | H7 | H17 | This study |
| **N46** | North Sea | H4 | H14 | This study |
| **N47** | North Sea | H7 | H17 | This study |
| **N48** | North Sea | H4 | H14 | This study |
| **N49** | North Sea |  | H17 | This study |
| **N50** | North Sea |  |  | This study |
| **N51** | North Sea | H7 | H17 | This study |
| **N52** | North Sea |  | H17 | This study |
| **N53** | North Sea | H7 | H17 | This study |
| **N54** | North Sea |  | H14 | This study |
| **N59** | North Sea | H7 | H17 | This study |
| **N60** | North Sea |  | H17 | This study |
| **N61** | North Sea |  | H17 | This study |
| **N62** | North Sea |  | H17 | This study |
| **N63** | North Sea |  | H17 | This study |
| **N64** | North Sea |  | H17 | This study |
| **N65** | North Sea | H10 | H17 | This study |
| **N81** | North Sea | H7 | H17 | This study |
| **N82** | North Sea | H7 | H17 | This study |
| **N83** | North Sea |  | H17 | This study |
| **N84** | North Sea | H7 | H17 | This study |
| **N85** | North Sea | H7 | H17 | This study |
| **N86** | North Sea | H7 | H17 | This study |
| **N87** | North Sea | H7 | H17 | This study |
| **N88** | North Sea | H7 | H17 | This study |
| **N89** | North Sea | H7 | H17 | This study |
| **N90** | North Sea | H7 | H17 | This study |
| **N91** | North Sea | H7 | H17 | This study |
| **N92** | North Sea | H7 | H17 | This study |
| **N93** | North Sea | H7 | H17 | This study |
| **N94** | North Sea | H7 | H17 | This study |
| **N95** | North Sea | H7 | H17 | This study |
| **N96** | North Sea | H7 | H17 | This study |
| **N97** | North Sea | H7 | H17 | This study |
| **N98** | North Sea |  | H17 | This study |
| **N99** | North Sea | H7 | H17 | This study |
| **N100** | North Sea | H7 | H17 | This study |
| **N101** | North Sea | H7 | H17 | This study |
| **N102** | North Sea |  | H17 | This study |
| **N103** | North Sea | H7 | H17 | This study |
| **N104** | North Sea | H7 | H17 | This study |
| **N105** | North Sea | H7 | H17 | This study |
| **N106** | North Sea | H7 | H17 | This study |
| **N107** | North Sea | H7 | H17 | This study |
| **N108** | North Sea | H7 | H17 | This study |
| **N109** | North Sea |  | H17 | This study |
| **O1** | East coast of Sardinia | H5 | H15 | This study |
| **O2** | East coast of Sardinia | H5 | H15 | This study |
| **O3** | East coast of Sardinia | H4 | H14 | This study |
| **O4** | East coast of Sardinia | H5 | H15 | This study |
| **O5** | East coast of Sardinia |  |  | This study |
| **O6** | East coast of Sardinia | H4 | H14 | This study |
| **O7** | East coast of Sardinia | H5 | H15 | This study |
| **O8** | East coast of Sardinia | H5 | H15 | This study |
| **O9** | East coast of Sardinia | H2 | H12 | This study |
| **O10** | East coast of Sardinia | H5 | H15 | This study |
| **O11** | East coast of Sardinia | H5 | H15 | This study |
| **O12** | East coast of Sardinia |  |  | This study |
| **O13** | East coast of Sardinia | H4 | H14 | This study |
| **O14** | East coast of Sardinia | H5 | H15 | This study |
| **O15** | East coast of Sardinia | H2 | H12 | This study |
| **O16** | East coast of Sardinia | H5 | H15 | This study |
| **O17** | East coast of Sardinia |  |  | This study |
| **O18** | East coast of Sardinia | H5 | H15 | This study |
| **O19** | East coast of Sardinia | H5 | H15 | This study |
| **O20** | East coast of Sardinia |  |  | This study |
| **O21** | East coast of Sardinia | H5 | H15 | This study |
| **O22** | East coast of Sardinia |  |  | This study |
| **O23** | East coast of Sardinia | H5 | H15 | This study |
| **O24** | East coast of Sardinia | H4 | H14 | This study |
| **O25** | East coast of Sardinia |  |  | This study |
| **O26** | East coast of Sardinia |  |  | This study |
| **O27** | East coast of Sardinia |  |  | This study |
| **O28** | East coast of Sardinia |  |  | This study |
| **O29** | East coast of Sardinia |  |  | This study |
| **O30** | East coast of Sardinia |  |  | This study |
| **S1** | Bay of Biscay |  | H17 | This study |
| **S2** | Bay of Biscay |  | H17 | This study |
| **S3** | Bay of Biscay |  |  | This study |
| **S4** | Bay of Biscay |  | H17 | This study |
| **S5** | Bay of Biscay |  |  | This study |
| **S6** | Bay of Biscay |  |  | This study |
| **S7** | Bay of Biscay |  |  | This study |
| **S8** | Bay of Biscay |  |  | This study |
| **S9** | Bay of Biscay |  | H17 | This study |
| **S10** | Bay of Biscay |  |  | This study |
| **S11** | Bay of Biscay | H7 | H17 | This study |
| **S12** | Bay of Biscay | H8 | H18 | This study |
| **S13** | Bay of Biscay |  | H17 | This study |
| **S14** | Bay of Biscay | H7 | H17 | This study |
| **S15** | Bay of Biscay | H7 | H17 | This study |
| **S16** | Bay of Biscay | H7 | H17 | This study |
| **S17** | Bay of Biscay | H7 | H17 | This study |
| **S18** | Bay of Biscay | H7 | H17 | This study |
| **S19** | Bay of Biscay |  |  | This study |
| **S20** | Bay of Biscay |  | H17 | This study |
| **S21** | Bay of Biscay | H7 | H17 | This study |
| **S22** | Bay of Biscay | H5 | H15 | This study |
| **S23** | Bay of Biscay |  |  | This study |
| **S24** | Bay of Biscay | H8 | H18 | This study |
| **W1** | West coast of Sardinia | H5 | H15 | This study |
| **W2** | West coast of Sardinia |  |  | This study |
| **W3** | West coast of Sardinia | H5 | H15 | This study |
| **W4** | West coast of Sardinia |  |  | This study |
| **W5** | West coast of Sardinia |  | H15 | This study |
| **W6** | West coast of Sardinia | H5 | H15 | This study |
| **W7** | West coast of Sardinia |  |  | This study |
| **W8** | West coast of Sardinia | H4 | H14 | This study |
| **W9** | West coast of Sardinia | H3 | H14 | This study |
| **W10** | West coast of Sardinia | H6 | H16 | This study |
| **W11** | West coast of Sardinia | H2 | H12 | This study |
| **W12** | West coast of Sardinia | H5 | H15 | This study |
| **W13** | West coast of Sardinia | H5 | H15 | This study |
| **W14** | West coast of Sardinia | H4 | H14 | This study |
| **W15** | West coast of Sardinia | H5 | H15 | This study |
| **W16** | West coast of Sardinia | H5 | H15 | This study |
| **W17** | West coast of Sardinia |  |  | This study |
| **W18** | West coast of Sardinia | H5 | H15 | This study |
| **W19** | West coast of Sardinia |  |  | This study |
| **W20** | West coast of Sardinia |  |  | This study |
| **W21** | West coast of Sardinia | H7 | H17 | This study |
| **AF075402** | English Channel |  | H17 | Anderson (2000) |
| **KF369140** | West coast of Portugal |  | H17 | Lobo et al. (2013) |
| **KF854077** | unknown | H7 | H17 | de Luna Sales et al. (2013) |
| **KF854078** | unknown | H7 | H17 | de Luna Sales et al. (2013) |
| **KM517903** | North Sea | H7 | H17 | Gebhardt & Knebelsberger (2015) |
| **KM517904** | North Sea | H7 | H17 | Gebhardt & Knebelsberger (2015) |
| **KM517905** | North Sea | H7 | H17 | Gebhardt & Knebelsberger (2015) |
| **KM517906** | North Sea | H7 | H17 | Gebhardt & Knebelsberger (2015) |
| **KM517907** | North Sea | H10 | H17 | Gebhardt & Knebelsberger (2015) |
| **KM517908** | North Sea | H7 | H17 | Gebhardt & Knebelsberger (2015) |
| **KM517909** | North Sea | H7 | H17 | Gebhardt & Knebelsberger (2015) |
| **KM517910** | North Sea | H7 | H17 | Gebhardt & Knebelsberger (2015) |
| **KM517911** | North Sea | H7 | H17 | Gebhardt & Knebelsberger (2015) |
| **KM517912** | North Sea | H7 | H17 | Gebhardt & Knebelsberger (2015) |
| **KM517913** | North Sea | H10 | H17 | Gebhardt & Knebelsberger (2015) |
| **KM517915** | North Sea | H7 | H17 | Gebhardt & Knebelsberger (2015) |
| **KM517916** | North Sea | H7 | H17 | Gebhardt & Knebelsberger (2015) |
| **KM517917** | North Sea | H7 | H17 | Gebhardt & Knebelsberger (2015) |
| **KM517918** | North Sea | H7 | H17 | Gebhardt & Knebelsberger (2015) |
| **KM517919** | North Sea | H7 | H17 | Gebhardt & Knebelsberger (2015) |
| **KM517920** | North Sea | H7 | H17 | Gebhardt & Knebelsberger (2015) |
| **KM517921** | North Sea | H7 | H17 | Gebhardt & Knebelsberger (2015) |
| **KM517922** | North Sea | H7 | H17 | Gebhardt & Knebelsberger (2015) |
| **KM517923** | North Sea | H7 | H17 | Gebhardt & Knebelsberger (2015) |
| **KM517924** | North Sea | H7 | H17 | Gebhardt & Knebelsberger (2015) |
| **KM517925** | North Sea | H7 | H17 | Gebhardt & Knebelsberger (2015) |
| **MH293018** | North East Atlantic | H7 | H17 | Tatulli et al. (2020) |
| **MH293090** | North East Atlantic |  | H17 | Tatulli et al. (2020) |
| **Z1** | Terceira Island, Azores (38°39'04.3"N 27°12'56.2"W). | H25 | H25 | This study |
| **Z2** | Terceira Island, Azores (38°39'04.3"N 27°12'56.2"W). | H25 | H25 | This study |
| **Z3** | Terceira Island, Azores (38°39'04.3"N 27°12'56.2"W). | H25 | H25 | This study |
| **Z4** | Terceira Island, Azores (38°39'04.3"N 27°12'56.2"W). | H25 | H25 | This study |
| **Z5** | Terceira Island, Azores (38°39'04.3"N 27°12'56.2"W). | H25 | H25 | This study |
| **Z7** | Terceira Island, Azores (38°39'04.3"N 27°12'56.2"W). | H25 | H25 | This study |
| **Z9** | Terceira Island, Azores (38°39'04.3"N 27°12'56.2"W). | H25 | H25 | This study |
| **Z10** | Terceira Island, Azores (38°39'04.3"N 27°12'56.2"W). | H25 | H25 | This study |
| **Z11** | Terceira Island, Azores (38°39'04.3"N 27°12'56.2"W). | H25 | H25 | This study |
| **Z12** | Terceira Island, Azores (38°39'04.3"N 27°12'56.2"W). | H25 | H25 | This study |
| **Z13** | Terceira Island, Azores (38°39'04.3"N 27°12'56.2"W). | H26 | H26 | This study |
| **Z14** | Terceira Island, Azores (38°39'04.3"N 27°12'56.2"W). | H22 | H22 | This study |
| **Z25** | Terceira Island, Azores (38°39'04.3"N 27°12'56.2"W). | H25 | H25 | This study |
| **Z29** | Terceira Island, Azores (38°39'04.3"N 27°12'56.2"W). | H25 | H25 | This study |
| **Z30** | Terceira Island, Azores (38°39'04.3"N 27°12'56.2"W). | H25 | H25 | This study |
| **Z31** | Terceira Island, Azores (38°39'04.3"N 27°12'56.2"W). | H21 | H21 | This study |
| **Z32** | Terceira Island, Azores (38°39'04.3"N 27°12'56.2"W). | H25 | H25 | This study |
| **Z33** | Terceira Island, Azores (38°39'04.3"N 27°12'56.2"W). | H25 | H25 | This study |
| **Z36** | Terceira Island, Azores (38°39'04.3"N 27°12'56.2"W). | H25 | H25 | This study |
| **Z37** | Terceira Island, Azores (38°39'04.3"N 27°12'56.2"W). | H24 | H25 | This study |
| **Z38** | Terceira Island, Azores (38°39'04.3"N 27°12'56.2"W). | H25 | H25 | This study |
| **Z39** | Terceira Island, Azores (38°39'04.3"N 27°12'56.2"W). | H25 | H25 | This study |
| **Z40** | Terceira Island, Azores (38°39'04.3"N 27°12'56.2"W). | H25 | H25 | This study |
| **Z41** | Terceira Island, Azores (38°39'04.3"N 27°12'56.2"W). | H25 | H25 | This study |
| **Z42** | Terceira Island, Azores (38°39'04.3"N 27°12'56.2"W). | H25 | H25 | This study |
| **Z43** | Terceira Island, Azores (38°39'04.3"N 27°12'56.2"W). | H25 | H25 | This study |
| **Z44** | Terceira Island, Azores (38°39'04.3"N 27°12'56.2"W). | H25 | H25 | This study |
| **Z48** | Terceira Island, Azores (38°39'04.3"N 27°12'56.2"W). | H25 | H25 | This study |
| **Z57** | Terceira Island, Azores (38°39'04.3"N 27°12'56.2"W). |  | H25 | This study |
| **Z58** | Terceira Island, Azores (38°39'04.3"N 27°12'56.2"W). |  | H25 | This study |
| **Z59** | Terceira Island, Azores (38°39'04.3"N 27°12'56.2"W). | H25 | H25 | This study |
| **Z60** | Terceira Island, Azores (38°39'04.3"N 27°12'56.2"W). |  | H25 | This study |
| **Z61** | Terceira Island, Azores (38°39'04.3"N 27°12'56.2"W). | H25 | H25 | This study |
| **Z62** | Terceira Island, Azores (38°39'04.3"N 27°12'56.2"W). | H25 | H25 | This study |
| **Z64** | Terceira Island, Azores (38°39'04.3"N 27°12'56.2"W). | H25 | H25 | This study |
| **Z65** | Terceira Island, Azores (38°39'04.3"N 27°12'56.2"W). | H25 | H25 | This study |
| **Z68** | Terceira Island, Azores (38°39'04.3"N 27°12'56.2"W). | H25 | H25 | This study |
| **Z69** | Terceira Island, Azores (38°39'04.3"N 27°12'56.2"W). | H25 | H25 | This study |
| **Z70** | Terceira Island, Azores (38°39'04.3"N 27°12'56.2"W). | H25 | H25 | This study |
| **Z71** | Terceira Island, Azores (38°39'04.3"N 27°12'56.2"W). |  | H25 | This study |
| **Z72** | Terceira Island, Azores (38°39'04.3"N 27°12'56.2"W). | H25 | H25 | This study |
| **Z74** | Terceira Island, Azores (38°39'04.3"N 27°12'56.2"W). |  | H25 | This study |
| **Z76** | Terceira Island, Azores (38°39'04.3"N 27°12'56.2"W). | H25 | H25 | This study |
| **Z78** | Terceira Island, Azores (38°39'04.3"N 27°12'56.2"W). | H25 | H25 | This study |
| **Z79** | Terceira Island, Azores (38°39'04.3"N 27°12'56.2"W). | H25 | H25 | This study |
| **Z79** | Terceira Island, Azores (38°39'04.3"N 27°12'56.2"W). | H25 | H25 | This study |
| **Z80** | Terceira Island, Azores (38°39'04.3"N 27°12'56.2"W). | H25 | H25 | This study |
| **AZB030-20** | Graciosa Island, Azores (38°39'04.3"N 27°12'56.2"W). | H25 | H25 | BoldSystems |
| **MT919766** | Portugal, Azores | H25 | H25 | Velasco et al. (2021) |
| **MT491734** | Portugal, Azores | H25 | H25 | Luz, A. & Keskin, E. (unpubl.) |

*GenBank Accession numbers for haplotype network with long sequences: H1:  OK135754, H2: OK135755, H3: OK135756, H4: OK135757, H5: OK135758, H6: OK135759, H7: OK135760, H8: OK135761, H9: OK135762, H10: OK135763, H21: OK135764, H22: OK135765, H23: OK135766, H24: OK135767, H25: OK135768, H26: OK135769.

**
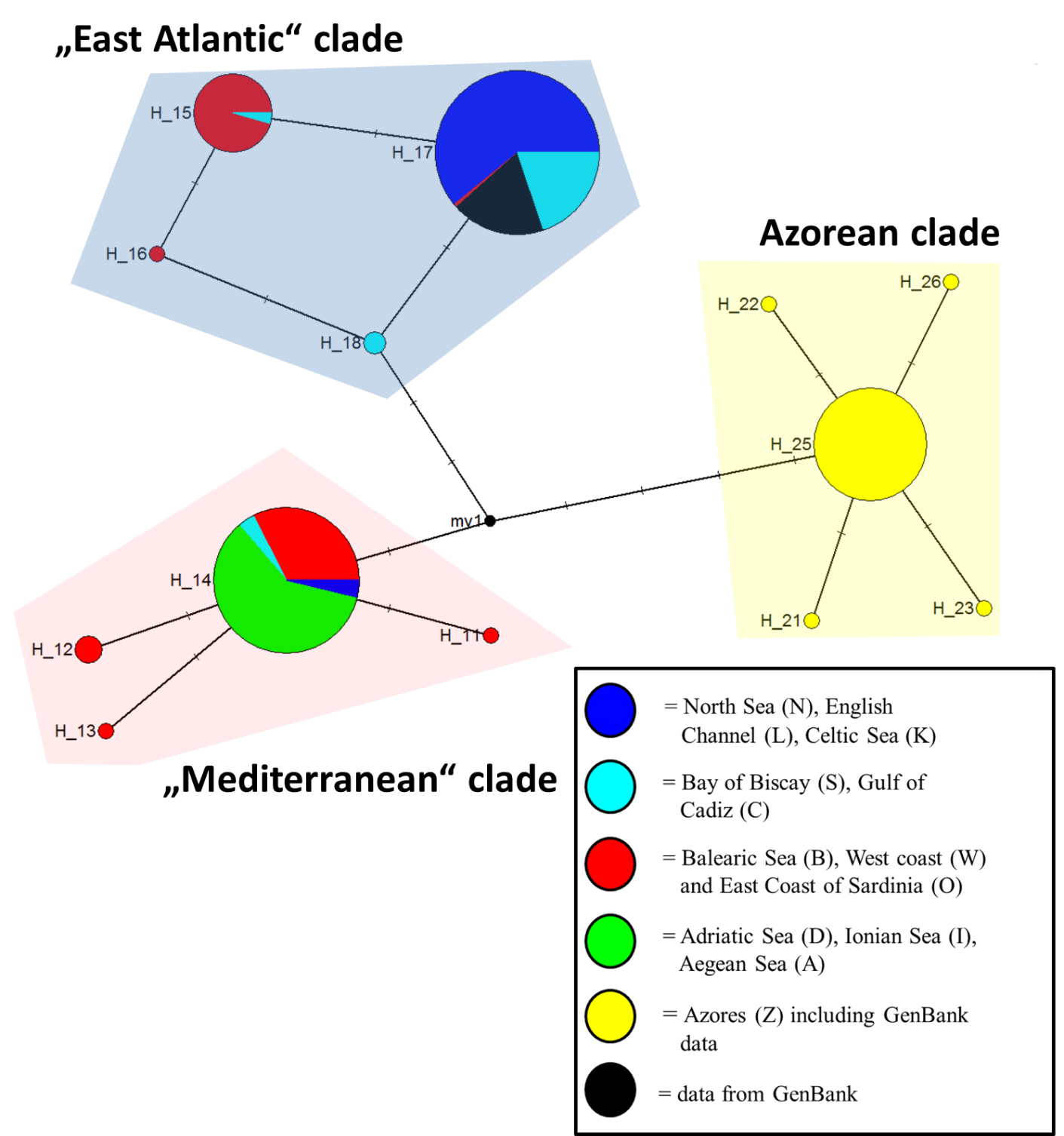
**

**Figure S1.** Median-Joining Haplotype network for *L. forbesii* (haplotypes 11-18, 21-23, 25, 26) representing 282 COI-sequence fragments (432 bp); black coloured GenBank data by various authors originate all from North East Atlantic individuals (Anderson (2000), Gebhardt & Knebelsberger (2015), Lobo et al. (2013), de Luna Sales et al. (2013), Tatulli et al. (2020)); yellow coloured including GenBank data (Velasco et al. (2021), Luz & Keskin (unpublished)) and BoldSystems data (**AZB030-20)**. The short sequences correspond to the long sequences from bp63 to bp494.


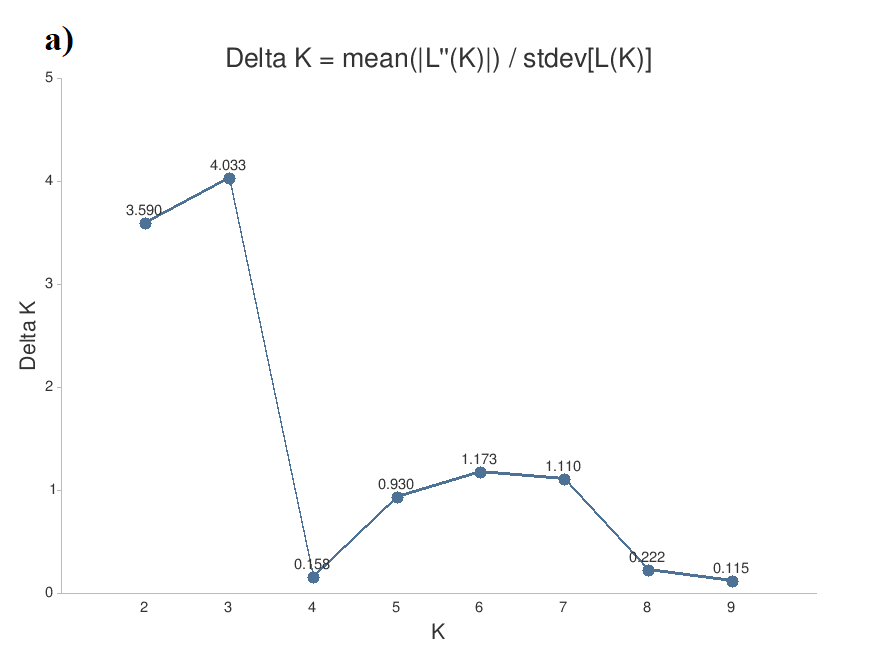

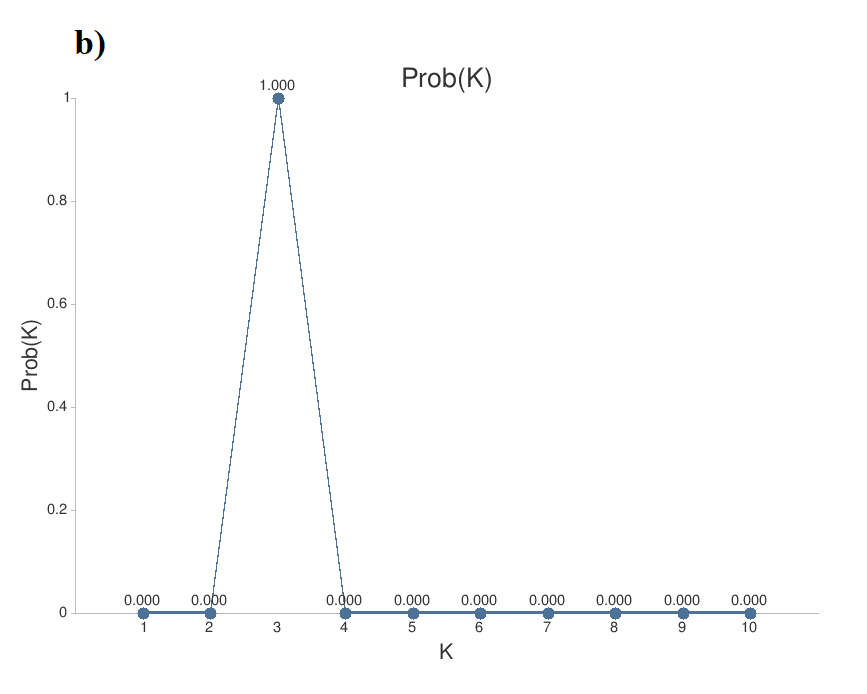


**Figure S2.** Results for best k by Evanno of the STRUCTURE Analysis; a) Delta K graph - optimal K by Evanno is: 3; b) Probability by K graph - using median values of Ln(Pr Data) the k for which Pr(K=k) is highest: 3.

**Figure S3**: The evolutionary history of Loliginidae was inferred by using the Maximum Likelihood method based on the General Time Reversible model (Nei & Kumar, 2000). The tree with the highest log likelihood (-2967.7473) is shown. The percentage of trees in which the associated taxa clustered together is shown next to the branches. A discrete Gamma distribution was used to model evolutionary rate differences among sites (16 categories (+G, parameter = 0.4934)). The rate variation model allowed for some sites to be evolutionarily invariable ([+I], 0.0000% sites). The tree is drawn to scale, with branch lengths measured in the number of substitutions per site. There is a total of 561 positions in the final dataset. Evolutionary analyses were conducted in MEGA6 (Tamura et al., 2013).

**
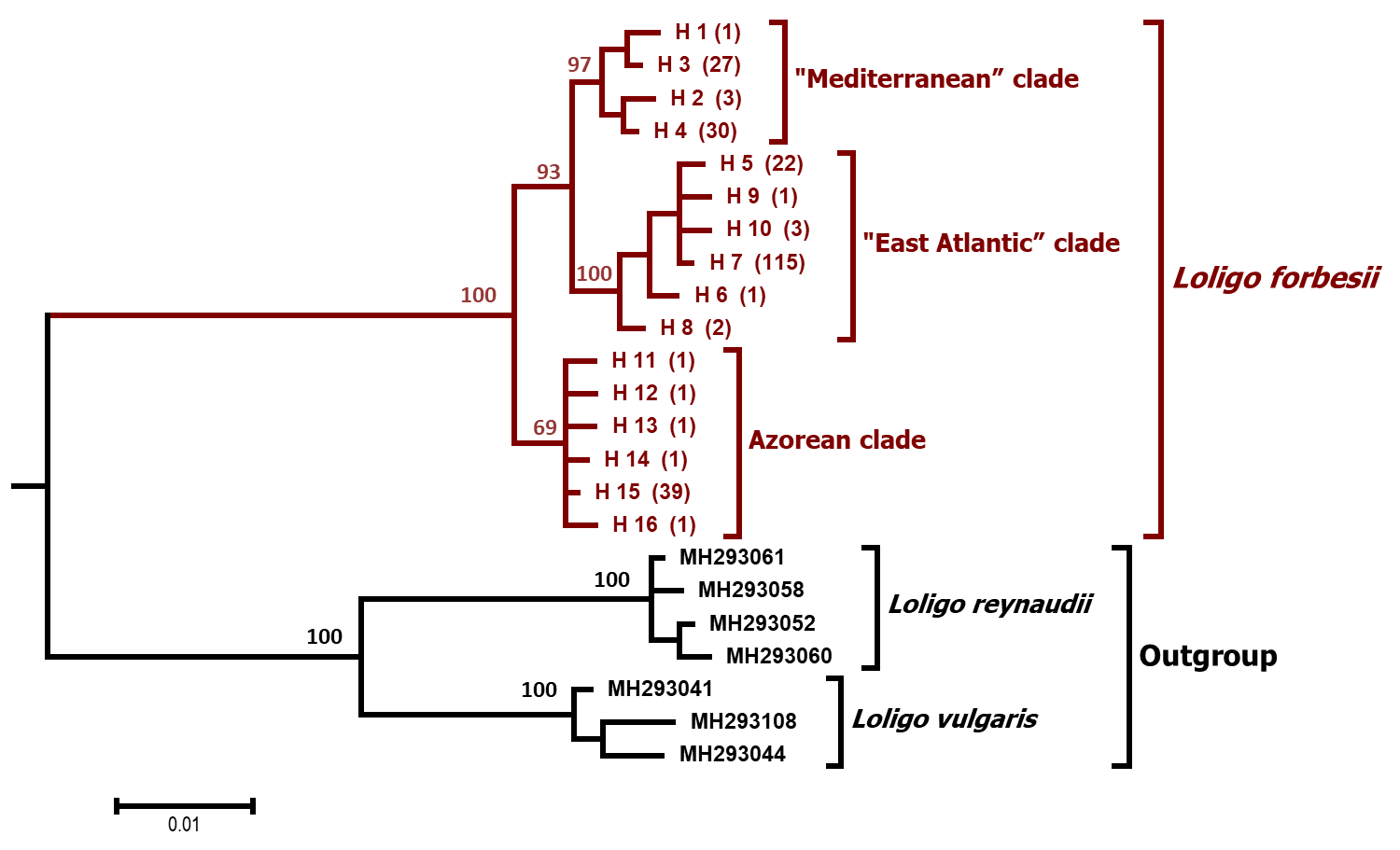
**

**Figure S4**. Phylogenetic tree based on 561 base pairs (bp) of the COI-sequences. The evolutionary history was inferred by using the K80+G model (Kimura, 1980). Bayesian posterior probabilities in percent (> 50%, BI) are given at the branches. The scale indicates sequence divergence in percent.


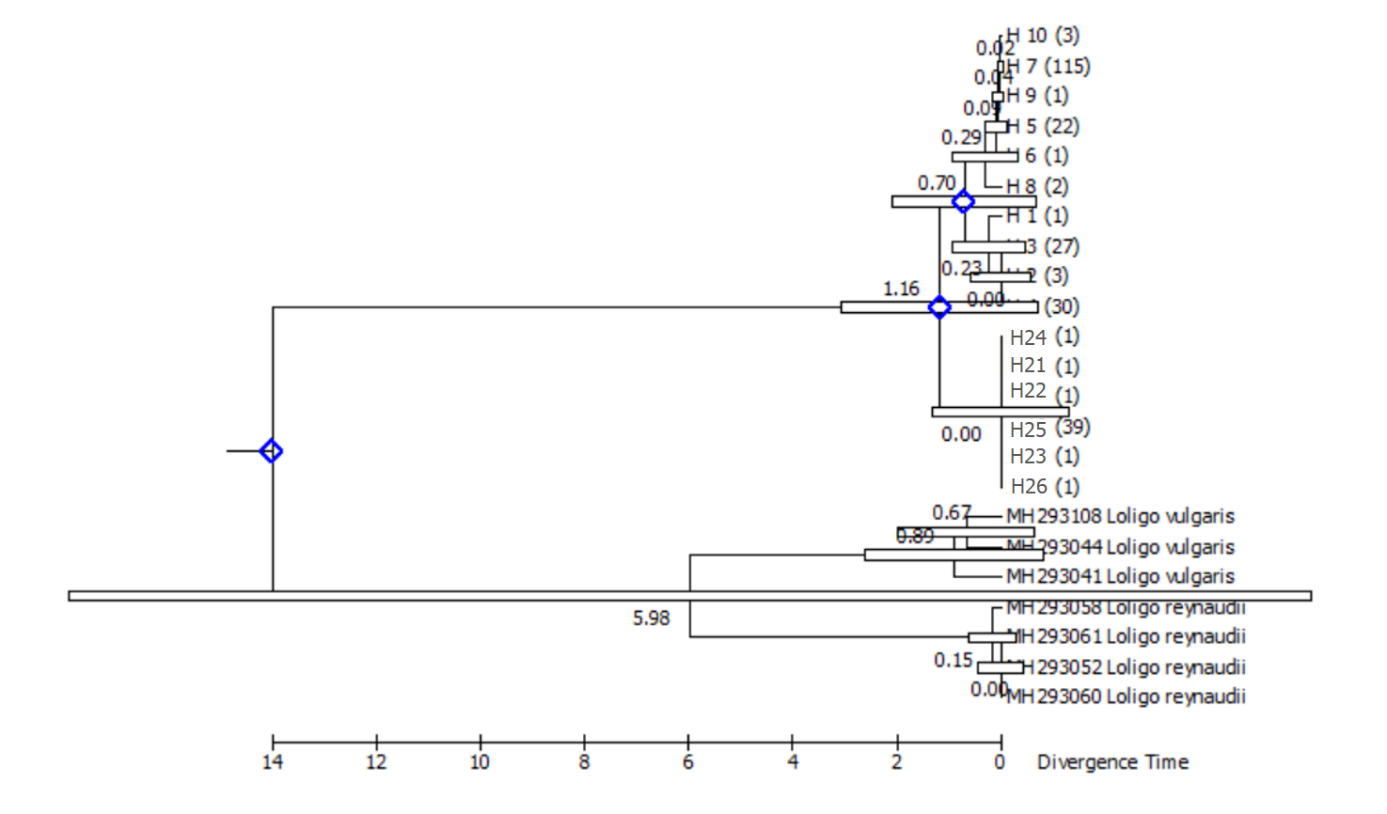


**Figure S5. Timetree analysis by Maximum Likelihood method;**

The timetree was generated using the RelTime method (Tamura et al., 2012). Divergence times (in million years ago) for all branching points in the user-supplied topology were calculated using the Maximum Likelihood method based on the Tamura-Nei model (Tamura & Nei, 1993). Relative times were optimized and converted to absolute divergence times (shown next to branching points) based on user-supplied calibration constraints. Bars around each node represent 95% confidence intervals which were computed using the method described in Tamura et al. (2013). The estimated log likelihood value of the topology shown is -1264.5023. A discrete Gamma distribution was used to model evolutionary rate differences among sites (5 categories (+*G*, parameter = 0.2727)). The tree is drawn to scale, with branch lengths measured in the relative number of substitutions per site. The analysis involved 23 nucleotide sequences. There was a total of 561 positions in the final dataset. Evolutionary analyses were conducted in MEGA6 (Tamura et al. 2013).

Calibration points were chosen as follows: separation of *L. forbesii* and sister taxon 11.0-16.0 million years ago (Ma), main branch of *L. forbesii* 1.0-5.5 Ma and establishing of the Mediterranean clade 0.1-1 Ma.

**References**

Anderson, F. E. (2000) Phylogeny and historical biogeography of the loliginid squids (Mollusca: Cephalopoda) based on mitochondrial DNA sequence data. *Mol. Phylogenet. Evol.* **15**, 191–214.

BoldSystems: <https://boldsystems.org/index.php/Public_RecordView?processid=AZB030-20>**; accessed 22-05-02.**

de Luna Sales, J. B. *et al.* (2013) New molecular phylogeny of the squids of the family Loliginidae with emphasis on the genus *Doryteuthis* Naef, 1912: Mitochondrial and nuclear sequences indicate the presence of cryptic species in the southern Atlantic Ocean. *Mol. Phylogenet. Evol.* **68**, 293–299.

Gebhardt, K. & Knebelsberger, T. (2015) Identification of cephalopod species from the North and Baltic Seas using morphology, COI and 18S rDNA sequences. *Helgol. Mar. Res.* **69**, 259–271.

Kimura, M. (1980) A simple method for estimating evolutionary rates of base substitutions through comparative studies of nucleotide sequences. *J. Mol. Evol.* **16**, 111–120.

Lobo, J. *et al.* (2013) Enhanced primers for amplification of DNA barcodes from a broad range of marine metazoans. *BMC Ecol.* **13**, 1–8.

Luz, A. & Keskin, E. (unpublished) Building Reference Library for Marine Fish Species of Azores Archipelago and Bio-monitoring via DNA Metabarcoding. <https://www.ncbi.nlm.nih.gov/nuccore/MT491734>

Nei, M. & Kumar, S. (2000) Molecular evolution and phylogenetics. *Oxford University Press*, USA.

Tamura, K. & Nei, M. (1993) Estimation of the number of nucleotide substitutions in the control region of mitochondrial DNA in humans and chimpanzees. *Mol. Biol. Evol.* **10**, 512–526.

Tamura, K. *et al.* (2012) Estimating divergence times in large molecular phylogenies. *Proc. Natl. Acad. Sci. U. S. A.* **109**, 19333–19338.

Tamura, K. *et al.* (2013) MEGA6: Molecular evolutionary genetics analysis version 6.0. *Mol. Biol. Evol.* **30**, 2725–2729.

Tatulli, G. *et al.* (2020) A Rapid Colorimetric Assay for On-Site Authentication of Cephalopod Species. *Biosensors* **10**, 3–10.

Velasco, A. *et al.* (2021) A new rapid method for the authentication of common octopus (*Octopus vulgaris*) in seafood products using recombinase polymerase amplification (rpa) and lateral flow assay (lfa). *Foods* **10**.
